# SHANK3 deficiency leads to myelin defects in the central and peripheral nervous system

**DOI:** 10.1007/s00018-022-04400-4

**Published:** 2022-06-20

**Authors:** Mariagiovanna Malara, Anne-Kathrin Lutz, Berra Incearap, Helen Friedericke Bauer, Silvia Cursano, Katrin Volbracht, Joanna Janina Lerner, Rakshita Pandey, Jan Philipp Delling, Valentin Ioannidis, Andrea Pérez Arévalo, Jaime Eugenin von Bernhardi, Michael Schön, Jürgen Bockmann, Leda Dimou, Tobias M. Boeckers

**Affiliations:** 1 Institute for Anatomy and Cell Biology, Ulm University, Albert-Einstein Allee 11, 89081 Ulm, Germany; 2 International Graduate School in Molecular Medicine, IGradU, 89081 Ulm, Germany; 3 Molecular and Translational Neuroscience, Department of Neurology, Ulm University, 89081 Ulm, Germany; 4 DZNE, Ulm Site, 89081 Ulm, Germany

**Keywords:** SHANK3, ASD, Myelin, hiPSCs

## Abstract

Mutations or deletions of the *SHANK3* gene are causative for Phelan–McDermid syndrome (PMDS), a syndromic form of autism spectrum disorders (ASDs). We analyzed *Shank3Δ11(−/−)* mice and organoids from PMDS individuals to study effects on myelin. SHANK3 was found to be expressed in oligodendrocytes and Schwann cells, and MRI analysis of *Shank3Δ11(−/−)* mice revealed a reduced volume of the corpus callosum as seen in PMDS patients. Myelin proteins including myelin basic protein showed significant temporal and regional differences with lower levels in the CNS but increased amounts in the PNS of *Shank3Δ11(−/−)* animals. Node, as well as paranode, lengths were increased and ultrastructural analysis revealed region-specific alterations of the myelin sheaths. In PMDS hiPSC-derived cerebral organoids we observed an altered number and delayed maturation of myelinating cells. These findings provide evidence that, in addition to a synaptic deregulation, impairment of myelin might profoundly contribute to the clinical manifestation of SHANK3 deficiency.

**Supplementary Information:**

The online version contains supplementary material available at 10.1007/s00018-022-04400-4.

## Introduction

Autism spectrum disorders (ASDs) are a group of neurodevelopmental conditions affecting approximately 1 in 68 people [1, 2]. The underlying causes are highly variable and include genetic as well as environmental factors [3, 4]. Nevertheless, the implicated biological processes in ASD patients seem to converge on synaptic alterations [5] and on changes in brain connectivity [6, 7]. Both hyper- and hypo-connectivity can be observed in ASD brains [8] along with changes in brain volume [9]. These pathological alterations seem to be highly dynamic, since some children with ASD were described to have an increased brain size while during adolescence and adulthood this is decreased [10, 11]. Furthermore, besides synaptic changes, a dysregulation of white matter has been described in multiple ASD patients [6, 12–14] and in ASD mouse models [15, 16].

One syndromic form of ASD is the Phelan–McDermid syndrome (PMDS), a rare neurodevelopmental disorder that accounts for 0.5% to 2.0% of ASD cases [17, 18]. The spectrum and severity of symptoms in affected patients are highly variable, but most individuals display global developmental delay, intellectual disability, speech impairment, neonatal hypotonia and autism-like behaviors [19]. PMDS is caused by heterozygous deletions in 22q13.3 where SH3 and multiple ankyrin repeat domains 3 ((SHANK3)/proline-rich synapse-associated protein 2 (ProSAP2)) is coded, or by genetic variations of *SHANK3* leading to its haploinsufficiency [20, 21]. *SHANK3* codes for a master scaffolding protein best known for its localization in the postsynaptic density (PSD) of excitatory synapses [22–24], where SHANK proteins link surface receptors, other PSD proteins and the actin cytoskeleton [25, 26]. In murine model systems reduced expression of SHANK3 leads to reduced number of dendrites, and impaired synaptic transmission and plasticity [27, 28].

Besides these synaptic changes, hippocampal and thalamic size have been reported to be changed in *Shank3Δ11(−/−)* mice using magnetic resonance imaging (MRI) analysis [29]. In addition, a clinical study by Jesse et al. (2019) has recently reported a broad range of intracerebral morphological abnormalities performing MRI studies in PMDS patients [30]. Thus, SHANK3 deficiency was attributed a white matter disease for the first time. A disturbance of white matter organization and volume has been observed in patients with PMDS, along with alterations in brain connectivity [30, 31].

This led us to speculate whether SHANK3 loss might change myelin. We used *Shank3Δ11(−/−)* mice [32] and cerebral organoids [33] from human-induced pluripotent stem cells of healthy individuals and PMDS patients to investigate SHANK3-associated changes. Since MRI analysis of the *Shank3Δ11(−/−)* mice revealed a significantly reduced volume of corpus callosum, we further examined the myelin composition and the expression of myelin proteins in young and adult mice (postnatal day P7, P21 and P140) in both the central (CNS) and peripheral nervous system (PNS). Lastly, our observations of alterations in myelin were confirmed by the analysis of hiPSC-derived cerebral organoids of PMDS patients. Based on these results, we propose that the symptoms observed in SHANK3-deficient patients are, at least in part, caused by altered myelin and do not exclusively arise from SHANK3 loss in neuronal synapses.

## Materials and methods

### Ethical statement

The human study was approved by the Ethics Committee of Ulm University (proposal no. 208/16 and 265/12). The experiments were conducted according to institutional and national guidelines and regulations. Prior to sampling, a written informed consent was provided by all the human donors and/or their legal guardians, as appropriate.

Ethical approval for the animal experiments was obtained by the review board of the Land Baden-Württemberg, Permit Number o.103-12 and z.103 TschB:W. The experiments were performed in compliance with the guidelines for the welfare of experimental animals issued by the Federal Government of Germany, the National Institutes of Health, and the Max Planck Society.

### Animal housing

Wildtype C57BL/6JRj mice (*Mus musculus*) were obtained from the animal research center of Ulm University. Knock-out Pro2 KO GVO (Shank3Δ11(−/−)) mice were housed as elucidated by [32] following heterozygous C57BL/6JRj mating. Animals were guarded in a pathogen-free facility under standard laboratory conditions with persistent food and water access, along with an average temperature of 22 °C and dark/light cycle as 12/12 rhythm. Mice were genotyped and grouped as wildtype *Shank3(*+ */* +*)*) or knock-out (*Shank3Δ11(−/−)*). For biochemical analysis 7-, 21- and 140-day-old male mice were used. For the MRI analysis four- and nine-week-old male and female animals were used.

### Volumetric analysis of corpus callosum from mouse MRI scans

Volumetric tissue analysis and data processing were performed by the in-house developed software package Tissue Classification Software (TCS) and has been described previously [29]. The analysis was performed blinded to genotype and all data were evaluated by the same investigator. Analysis was performed in coronal planes. The corpus callosum and Bregma coordinates were identified manually according to the Allen Brain Atlas [34] and a mouse brain atlas [35]. The corpus callosum was analyzed for both hemispheres.

### Cultivation of hiPSCs

All iPSC lines of this study have been reprogrammed from human keratinocytes. Seven different iPS cell lines were used for this study: three from healthy individuals, three from SHANK3 PMDS patients and one CRISPR/Cas9 engineered line carrying a *SHANK3* point mutation. They have previously been characterized for pluripotency and have already been published [36]. hiPSC colonies were passaged by treating them with dispase (Stemcell Technologies) for two minutes. They were washed twice with DMEM/F12 with GlutaMAX (Gibco), mechanically lifted and plated and maintained on feeder-free plates coated with hESC qualified matrigel (Corning) in mTeSR1 (Stemcell Technologies) at 37 °C, 5% CO_2_ and 5% O_2_. Cells were scratched, washed and fed daily.

### hiPSC-derived cerebral organoids

Cerebral organoids were generated following published protocols [33]. They were kept in culture for 60 or 110 days. Fixation was performed using 4% PFA (Merck)/0.1 M sucrose (Roth) in 1 × DPBS (Gibco) for 1 h and then cerebral organoids were washed three times for 10 min with 1 × DPBS. Collected tissue was cryoprotected with 30% sucrose in 1 × DPBS at 4 °C overnight. Tissues were afterward embedded in 7.5% gelatin (Sigma)/10% sucrose and cut into 20 µm thick sections, directly collected on microscopy slides using a Leica CM1950 cryostat. Immunohistochemistry was performed by treating the tissue with pre-warm antigen retrieval 0.1 M citrate buffer (pH 6) at room temperature (RT) for 20 min (for anti-MBP (1:300, Abcam)) or organoids were immediately blocked with 10% goat-serum (Millipore), and 0.1% Triton X-100 (Roche) diluted in 1 × DPBS/blocking solution at RT for 2 h. Sections were then immuno-labeled with corresponding antibodies at 4 °C for 24 h. Air–liquid interface cerebral organoids (ALI-COs) were generated following published protocols [37]. They were cultured for 60 days as cerebral organoid and then sectioned using the Leica VT1200S Vibratome to obtain organotypic sections of 400 µM thickness. Sections were maintained as slice culture for another 60 days.

### Cultivation of primary oligodendrocytes

Purified cultures of oligodendrocytes were prepared from total cortex of P0-P2 of wildtype *Shank3(*+ */* +*)* mouse pups. To obtain a mixed glial culture, the tissue was dissociated following published protocols [38] and the cells were seeded in 75 cm^2^ flasks coated with poly-l-lysine (0.1 mg/ml, Sigma) diluted in borate buffer (Thermo). OPC medium (DMEM with L-glutamine, 4.5 g/l glucose and sodium pyruvate, 10% FBS, 1% Pen-Strep) was changed every two to three days until a confluent monolayer has grown. Three days before purification the OPC medium was supplemented with bFGF (PeproTech Inc.) and PDGF-aa (R&D). After 15–20 days in culture, a monolayer mixture of astrocytes, microglia and OPCs was obtained. Flasks were shaken for 20 h at 250 rpm and 37 °C. Subsequently, the medium containing the detached OPCs was collected, and the cells were seeded at a density of 2.5 × 10^4^ cells/cm^2^ onto poly-l-lysine-coated 12 mm wide glass coverslips (for immunocytochemistry experiments) and 12 × 10^4^ cells/cm^2^ into poly-l-lysine-coated 6-well plates (for Western Blot analysis). On day 1 after purification, a full medium change was performed with proliferation medium (DMEM + pyruvate, 2 mM l-glutamine (Thermo), 100 µg/ml apo-transferrin, 100 µg/ml BSA, 40 µg/ml sodium selenite (Sigma), 60 ng/ml progesterone (Sigma), 16 µg/ml putrescine (Sigma), 5 µg/ml insulin (Merck), 10 ng/ml bFGF, 10 ng/ml PDGF-aa, 30 ng/ml T3, 40 ng/ml Thyroxine (Merck)). On day 2, proliferation medium was replaced by differentiation medium (DMEM + pyruvate, 2 mM L-glutamine (Thermo), FBS (0,5% v/v), 100 µg/ml apo-transferrin, 100 µg/ml BSA, 40 µg/ml sodium selenite (Sigma), 60 ng/ml progesterone (Sigma), 16 µg/ml putrescine (Sigma), 5 µg/ml insulin (Merck), 30 ng/ml T3, 40 ng/ml thyroxine (Merck)). After 6 days in differentiation medium, cells were ready for immunocytochemistry and lysates.

### Cultivation of Schwann cells

Commercial primary mouse Schwann cells (ScienCell Research Laboratories, #M1700-57) isolated from 8-day-old C57BL/6 mice were used. They were passaged by incubating them with TrypLE (Gibco) for one minute at 37 °C. The flask was then softly knocked to detach the cells from the surface, 8 ml 1 × DPBS (Gibco) were added, and cells were transferred into a falcon. Cells were centrifuged at 1000 rpm for 5 min, re-suspended in Schwann cell medium (ScienCell Research Laboratories, #1701) and were cultured on poly-l-lysine-hydrobromide (Sigma, 1 mg/ml) coated plates at 37 °C. At 80% confluency, cells were stained or used for Western Blot lysates.

### Immunocytochemistry

The cells were washed with DPBS, fixed with 4% PFA (Merck)/0.1 M sucrose (Roth) in 1 × DPBS (Gibco) for 20 min at RT and washed three times for 5 min with 1 × DPBS. They were permeabilized with 0.2% Triton X-100 (Roche) diluted in 1 × DPBS for 10 min at RT. After blocking with 10% goat-serum (Millipore), 5% FBS (Gibco) in 1 × DPBS at RT for 4 h, the primary antibodies were incubated at 4 °C for 24 h. The following antibodies were used: anti-SHANK3 (rb, 1:500, homemade [32]), anti-MBP (ms, 1:200, BioLegend), anti-O4 (ms, 1:500 R&D). After a short, 5-min, 10-min and 20-min washing steps, the secondary antibodies (Alexa Fluor 488 goat anti-mouse, Alexa Fluor 568 goat anti-rabbit, Invitrogen) were diluted 1:1000 in 1 × DPBS (Gibco) and incubated light protected at RT for 1 h. Washing was performed as described previously and coverslips were mounted with ProLong Gold Antifade reagent with DAPI (Invitrogen).

### Immunohistochemistry

ALI-COs were washed with DPBS and fixed with 4% PFA (Merck)/0.1 M sucrose (Roth) in 1 × DPBS (Gibco) overnight at 4 °C. After the fixation, the individual sections were removed from the cell culture inserts, transferred to a 24-well tissue culture plate, and blocked with 5% FBS (Gibco) and 0.5% Triton X-100 (Roche) diluted in 1 × DPBS at RT for a minimum of 4 h. Sections were then immuno-labeled with corresponding antibodies at 4 °C for 48 h. The following primary antibodies were used: anti-MBP (rb, 1:300, Abcam), anti-NeuN (rb, 1:500, Abcam), anti-SHANK3 (rb, 1:500, homemade [32]), anti-CC1 (ms, 1:500, Merck OP80), anti-CNPase (ms, 1:200, Abcam).

For immunostaining of the brain, spinal cord and sciatic nerve, mice were anesthetized by intraperitoneal injection of ketamine (WDT, 10%) and xylazine (Rompun 2%, Bayer, 20 µl/g body weight) in saline solution. Mice were trans-cardially perfused with 4% PFA (pH 7.4, Merck) in DPBS. Collected tissue was post-fixed in 4% PFA overnight and cryoprotected with 30% sucrose in 1 × DPBS at 4 °C for 18 h. Tissues were afterward lodged in O.C.T. (Tissue-Tek), cut in transversal or longitudinal 16 µm (sciatic nerve), 20 µm (spinal cord) or 40 µm thick sections (brain) using a Leica CM1950 cryostat. Sciatic nerve sections were collected directly on microscopy slides, while free-floating brain and spinal cord slices were suspended first in 1 × DPBS, then in cryoprotectant (50% 1 × DPBS, 30% ethylene glycol, 20% glycerol). To use the sections, the cryoprotectant was removed and sections were washed three times with DPBS. The procedure was followed either by antigen retrieval with pre-warm 0.1 M citrate buffer (pH 6) at room temperature (RT) for 20 min (for Anti-Caspr (1:200, NeuroMab) and Anti-Kv1.2 (1:200, NeuroMab)) or were immediately blocked with 10% goat-serum (Millipore), 5% FBS (Gibco) and 0.1% Triton X-100 (Roche) diluted in 1 × DPBS/blocking solution at RT for 4 h. Sections were then immuno-labeled with corresponding antibodies at 4 °C for 48 h. The following primary antibodies were used: anti-MBP (ms, 1:250, BioLegend), anti-MBP (rt, 1:200, Millipore), anti-NFH (ck, 1:1000, antibodies-online ABIN361351), anti-SHANK3 (rb, 1:500, homemade[32]), anti-CASPR (ms, 1:200, NeuroMab), anti-Kv1.2 (ms, 1:200, NeuroMab), anti-CC1 (ms, 1:250, Merck OP80), anti-CNPase (ms, 1:250, Abcam), anti-MPZ (ck, 1:500, Aves). The tissue was gently washed four times with 1 × DPBS: short, 5 min, 10 min and 20 min. Secondary antibodies Alexa Fluor 488 donkey anti-rabbit or Alexa Fluor 488 goat anti-rabbit, Alexa Fluor 594 donkey anti-chicken or Alexa Fluor 568 goat anti-mouse, Alexa Fluor 647 donkey anti-mouse or Alexa Fluor 647 goat anti-chicken were obtained from Invitrogen, Jackson ImmunoResearch Laboratories or Chromotek (ms568 IgG1 and ms647 IgG2), respectively. The dilution was 1:1000 in blocking solution and incubation was light protected at RT for 2 h. Following the same washing steps, tissue sections were mounted with ProLong Gold Antifade reagent with DAPI (Invitrogen). Overview images were acquired using a Leica DMi8 microscope. Detailed images were acquired with a resolution of 1024 × 1024 pixels by utilizing the Leica TCS SPE II confocal microscope (Wetzlar, Germany). For white and gray matter thickness analysis in MBP staining, a Keyence BZ-X810 fluorescence microscope was used.

### Expansion microscopy

Mouse brain sections undergoing expansion microscopy were obtained from *Shank3(*+ */* +*)* mice as described in the ‘immunohistochemistry’ chapter. After removal of cryoprotectant, sections were washed for three times with DPBS and incubated with primary antibodies diluted in blocking solution (3% BSA and 0.3% Triton X-100 in DPBS) twice, once pre-expansion and post-denaturation. Both times, the samples were incubated over the weekend at 4 °C with primary antibodies anti-SHANK3 (rb 1:250 homemade), anti-MBP (ms, 1:100, BioLegend), anti-NFH (ck, 1:2500, antibodies-online). Secondary antibody incubation was performed overnight at room temperature and a dilution of 1:200, but only post-denaturation. After the first primary antibody incubation, the TREx protocol was applied with minor adjustments to expand the samples [39]. Briefly, brain slices were treated with 10 µg/ml acryloyl X-SE in PBS−/− overnight at room temperature. The gelation solution contained 1.1 M sodium acrylate, 2.0 M acrylamide, 50 ppm *N*,*N*′-methylenebisacrylamide and PBS−/− (1x) once the polymerization was initiated by the addition of TEMED (1.5 ppt) and APS (1.5 ppt). The polymerization process was slowed by 4-Hydroxy-TEMPO (22 ppm) to allow for an extended free-floating incubation time of 45 min in activated gelation solution, before mounting the slices into the gelation chamber and allow full polymerization for 1 h at 37 °C. Then, slices were recovered into digestion buffer (50 mM Tris-BASE, 200 mM NaCl, 200 mM SDS in ddH2O) and incubated for 3 h at 80 °C in a Thermo-Block. After denaturation, the samples did undergo a second round of immunofluorescent staining as mentioned above. The stained gels were then incubated with DAPI (Roth, 6335.1, 1:50.000) for 5 min at room temperature. Afterward the samples were washed 4–5 times in ddH2O and stored at 4 °C overnight to complete the expansion of the gel.

For imaging, the region of interest was cut from the gel with a scalpel and mounted onto a #1.5H chambered cover glass (Cellvis). Images were acquired using a Leica TCS SPE II confocal microscope (Wetzlar, Germany) equipped with a HC PL APO 63x/1,30 GLYC CORR CS2 glycerol-immersion objective. Images were deconvolved using Huygens Professional (SVI) version 21.04 and further processed using FIJI (ImageJ 1.53f51).

### FluoroMyelin

Sciatic nerve cryosections were post-fixed with 4% PFA (Merck)/0.1 M sucrose (Roth) in 1 × DPBS (Gibco) for 15 min. Then they were permeabilized with 0.2% Triton X-100 (Roche) in 1 × DPBS for 1 h. Free-floating 40 µm thick brain as well as 20 µm spinal cord slices were permeabilized for 3 h. Afterward, the tissues were stained with FluoroMyelin Red (Invitrogen) diluted 1:300 in 1 × DPBS for 3 or 1 h, respectively. After washing 3 times with 1 × DPBS for 10 min each, the sections were mounted with ProLong Gold Antifade reagent with DAPI (Invitrogen). Overview images were acquired using a Leica DMi8 microscope. Detail images were acquired with a resolution of 1024 × 1024 pixels using the Leica TCS SPE II confocal microscope (Wetzlar, Germany).

### Western Blot

Brain, cervical spinal cord, and sciatic nerve were mechanically homogenized in modified RIPA buffer (10 mM Tris–HCl pH 7.4 (AppliChem), 0.1% sodium dodecyl sulfate (SDS, Roth), 1% Triton X-100 (Roche), 1% sodium-deoxycholate (Merck), 5 mM EDTA (Sigma), protease/phosphatase inhibitor (Roche)) utilizing an electric tissue grinder and were lysed on ice for 15 min. The lysate was sonicated 10 times for 10 s and further incubated on ice for 30 min. Lysates were clarified by centrifuging at 13,000 rpm at 4 °C for 10 min. Protein concentration was determined via Bradford assay. Therefore, 20 µl of 150 mM NaCl (Merck), 2 µl of the vortexed sample and 200 µl Bradford solution ((ethanol (95%, Roth), phosphoric acid (85%, VWR), Serva Blue (Serva) solved in distilled water) were pipetted as duplicates into a 96-well plate (Sarstedt). The plate was placed into a microplate reader and the absorbance was measured at 595 nm. The measured values were used to calculate the protein concentration of the sample and were prepared in water and sodium dodecyl sulfate ACS reagent (SDS, ≥ 99.0%, Roth) according to the desired load. Samples were boiled at 95 °C for 5 min. Gel electrophoresis was performed at 90 V for about 15 min and then at 110 V for further 45 min. The protein was blotted onto a nitrocellulose membrane by a Trans-Blot® Turbo™ Transfer System (BioRad), the membranes were blocked with 5% skim milk powder (Sigma) in Tris-buffered saline with 0.1% TWEEN-20 (TBST). The membranes were incubated with primary antibodies at 4 °C overnight. The following primary antibodies were used: anti-SHANK3 (rb, 1:500, homemade[32]), anti-MBP (ms, 1:500, BioLegend), anti-β-ACTIN (ms, 1: 250,000, Sigma), anti-Olig2 (1:500, Chemicon). The next day, the membranes were washed three times for 20 min with 0.1% TBST and were incubated with HRP-conjugated secondary antibodies (Dako) at RT for 1 h. Membranes were washed three times for 20 min with 0.1% TBST. Protein was visualized using the Chemiluminescent Western Blot Reagent (Thermo Fischer). Bands were analyzed using Gel-analyzer Software 2010a.

### Electron microscopy

Following cervical dislocation, brain, spinal cord and sciatic nerve were dissected from the mice and fixed in pre-cooled 2% paraformaldehyde (PFA, pH 7.3, Merck), 2.5% glutaraldehyde (GA, Plano Agar), 0.1 M Sorensen’s phosphate buffer, 1% saccharose at 4 °C for 24 h. The tissues were cut into small fragments and further fixed with 2.5% GA, 0.1 M Sorensen’s phosphate buffer, 1% saccharose at 4 °C for 24 h. Afterward, the tissues were post-fixed in 2% aqueous osmium tetroxide (Fluka), followed by stepwise dehydration of the samples with propanol. Thereafter, the samples were embedded in epoxy resin (Fluka). Semi-thin sections of 500 nm were cut and post-stained with 1% toluidine blue (Fluka) and 1% Na–borate (Sigma) in water. Resin blocks were trimmed, followed by cutting into ultra-thin 80 nm sections and placing them on copper grids 300mesh (Plano). Finally, the sections were contrasted with 0.3% lead citrate (Plano Agar). Images were taken using a JEOL 1400 Transmission Electron Microscope (TEM) and a Veleta camera (Olympus).

### Image analysis

For thickness measurements in cortex and corpus callosum in MBP stained slices, distances were measured from the mid-line of the brain as indicated in the picture.

To ascertain FluoroMyelin, MBP, MPZ and SHANK3 intensity of brain, spinal cord, and the sciatic nerve, 5–6 confocal images per animal were analyzed measuring the mean gray value of the positive area per picture (sciatic nerve) or region of interest (ROI size brain: 64 × 64 μm, ROI size spinal cord: 44 × 44 μm) using Fiji ImageJ (Wayne Rasband, National Institutes of Health, USA). The maximum projection of all pictures was created in Fiji ImageJ. Within the NFH and MBP channel, a threshold, dependent on the intensity histogram and covering all the signal was set and a corresponding mask created. These masks of the first channel were overlapped to the SHANK3 channel, the second channel of interest, to determine the SHANK3 intensity only in the NFH-positive or MBP-positive area, respectively. Images of one experiment containing all experimental groups were always imaged together to ensure comparability. Since the absolute intensities varied between experiments, data were normalized to the mean of all data points obtained from the same experiment. One experiment included either sections of three *Shank3(*+ */* +*)* and three *Shank3Δ11(−/−)* animals or sections of one *Shank3(*+ */* +*)* and *Shank3Δ11(−/−)* animal each.

To assess the complexity of the MBP staining in cortex, the number of intersections (intersection density (100 × 100px)) was analyzed using the ImageJ plugin “Diameter J” as described previously [40].

Paranode, node and juxta-paranode analyses of transversal brain and longitudinal spinal cord sections were analyzed by acquiring six confocal images per animal. To evaluate paranode and node length the “straight line” tool was applied using Fiji ImageJ. Nine CASPR/Kv1.2 and node segments were analyzed per image. Each Kv1.2 fragment of a CASPR segment was counted and classified as adjacent or overlap.

Paranode, node and juxta-paranode analyses of the sciatic nerve were conducted by taking 3–6 confocal pictures per animal. To measure the CASPR and the node length the “straight line” tool was applied using Fiji ImageJ. For P7: 30–180 CASPR/ Kv1.2 and 15–90 node segments per mouse, 4–86 CASPR/Kv1.2 structures and 2–43 node segments per slice; P21: 42–166 CASPR/Kv1.2 and 21–83 node segments per mouse, 20–46 CASPR/ Kv1.2 structures and 10–23 node segment per slice and for P140: 64–150 CASPR/Kv1.2 and 32–75 node segment per mouse, 10–38 CASPR/Kv1.2 structures and 5–19 node segment per slice were analyzed, respectively. Each Kv1.2 fragment of a CASPR segment was counted and classified as adjacent or overlap.

MBP and NeuN positive cells in cerebral organoids were counted considering three regions of interest per image. Values were set relative to the total amount of DAPI nuclei per area.

MBP (cerebral organoids), CC1 and CNP (slice organoids) intensity in hiPSC-derived cerebral organoids were analyzed by measuring the mean gray value of the positive area per region of interest using Fiji ImageJ. Within the CC1 channel (slice organoids) a threshold was applied, creating the respective mask. Thus, SHANK3 intensity was analyzed only in the CC1-positive area. Data were normalized to the mean of controls. Overview tile scan images were acquired on a Leica DMi8 microscope and high-resolution images were obtained using a Leica TCS SPE II confocal microscope (Wetzlar, Germany).

Electron microscopic images from brain, spinal cord and sciatic nerve were analyzed using the “free hand selections” tool in Fiji (ImageJ) to measure the area of an axon or the entire fiber. From these values the axon diameter and g ratio (≥ 100 axon–myelin units per animal) were calculated in Microsoft Excel.

For analysis of expansion microscopy, we analyzed transversal axonal section using Fiji image J. Three consecutive steps were collapsed as maximum intensity projection. The outer-most layer of the MBP signal was surrounded and selected as ROI to measure one complete fiber at a time. Within that ROI, the integrated intensity of the SHANK3 channel was measured. This value of SHANK3 content was set to 100%. Then, the area covered by the MBP signal was saved as a selection. This selection was put on the SHANK3 channel and the integrated density of SHANK3 was measured within this MBP mask. The SHANK3 content localizing within the MBP mask was calculated as percentage of SHANK3 in the complete fiber.

The processed measurements were plotted with GraphPad Prism 8.

### Statistical analysis

Statistical analyses of data and graphs were performed using GraphPad Prism 8 Software and/or Microsoft Excel. Data display the mean ± Standard Error of Mean (SEM). Data were checked for normality using Shapiro–Wilk normality test. Two-tailed Student’s *t* test (parametric data) or Mann–Whitney test (non-parametric data) were used to compare two groups. Categorized data with more than two classes were tested with Fisher’s exact test using R version 4.1.0 and rstatix package. Significance levels (p values) were set to 0.05 (*p* ≤ 0.05*, *p* ≤ 0.01**, *p* ≤ 0.001***, *p* ≤ 0.001****) with 95% confidence interval.

## Results

### SHANK3 loss leads to myelin alterations in the central nervous system of *Shank3Δ11(−/−)* mice

**Fig. 1:**
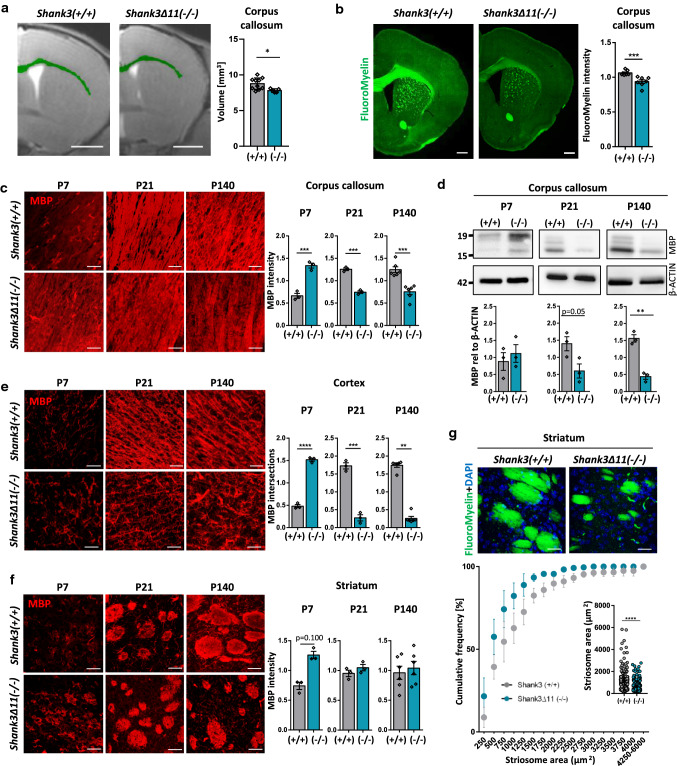
White matter and MBP expression are changed in the brain of *Shank3Δ11(−/−)* mice. **a** MRI analysis of corpus callosum volume in 9-week-old male *Shank3(*+ */* +*)* and *Shank3Δ11(−/−)* animals. Scale bar 2 mm. *n* = 10 + / + , n = 5 −/− animals. Mean ± SEM. Student’s Unpaired *t* test, *p* = 0.0211. **b** FluoroMyelin intensity analysis in corpus callosum of 140-day-old *Shank3(*+ */* +*)* and *Shank3Δ11(−/−)* animals. Scale bar 500 μm. *n* = 7 animals. Mean ± SEM. Mann–Whitney test, *p* = 0.0006. **c** MBP IHC and intensity analysis in corpus callosum of 7-, 21- and 140-day-old *Shank3(*+ */* +*)* and *Shank3Δ11(−/−)* animals. Scale bar 30 μm. *n* = 3 animals, P140 n = 6. Mean ± SEM. Student’s Unpaired *t* test, P7 *p* = 0.0010, P21 *p* = 0.0002, P140 *p* = 0.0003. **d** Western Blot analysis of MBP and β-ACTIN in corpus callosum of 7-, 21- and 140-day-old *Shank3(*+ */* +*)* and *Shank3Δ11(−/−)* animals. *n* = 3 animals. Mean ± SEM. Student’s Unpaired *t* test, P7 *p* = 0.5564, P21 *p* = 0.0526, P140 *p* = 0.0011. **e** MBP IHC and analysis of number of intersections in cortex of 7-, 21- and 140-day-old *Shank3(*+ */* +*)* and *Shank3Δ11(−/−)* animals. Scale bar 30 μm. n = 3 animals, P140 n = 6. Mean ± SEM. Student’s Unpaired *t* test, P7 *p* < 0.0001, P21 *p* = 0.0003, Mann–Whitney test P140 *p* = 0.0022. **f** MBP IHC and intensity analysis in striatum of 7-, 21- and 140-day-old *Shank3(*+ */* +*)* and *Shank3Δ11(−/−)* animals. Scale bar 30 μm. *n* = 3 animals, P140 *n* = 6. Mean ± SEM. Mann–Whitney test P7 *p* = 0.100, Student’s Unpaired *t* test, P21 *p* = 0.2726, P140 *p* = 0.6264. **g** FluoroMyelin and DAPI staining in striatum of 140-day-old *Shank3(*+ */* +*)* and *Shank3Δ11(−/−)* animals and analysis of striosome area. Scale bar 30 μm. *n* = 4 striosomes collected from 4 animals. Mean ± SEM. Student’s Unpaired *t* test, *p* < 0.0001

To assess potential white matter alterations in *Shank3Δ11(−/−)* mice, we performed structural MRI analysis and found a significant decrease in the volume of corpus callosum compared to *Shank3(*+ */* +*)* mice (Fig. 1a). Four- and nine-week-old animals were analyzed and a decrease in the volume of corpus callosum was consistently found in both genders and hemispheres (Suppl. Figure 1a, b). The overall brain volume was decreased in four-week-old but not in nine-week-old animals (Suppl. Figure 1c). A specific reduction of the white matter was further confirmed in immuno-stained slices against myelin basic protein (MBP) by measuring the thickness of the cortex and corpus callosum at discrete mediolateral positions starting from the mid-line and moving laterally in intervals of 200 μm (Suppl. Figure 1d). To see if those changes correlate with SHANK3 loss, Western Blot analysis of protein lysates of the corpus callosum was performed and indeed SHANK3 expression was reduced in *Shank3Δ11(−/−)* 140-day-old (P140) mice (Suppl. Figure 2a). To specifically assess the myelin in the corpus callosum, brain tissue was stained with FluoroMyelin dye, and the staining intensity was found significantly reduced in *Shank3Δ11(−/−)* mice (Fig. 1b). FluoroMyelin intensity was also reduced in cortex but not in striatum (Suppl. Figure 2b). Considering SHANK3-ASD as a neurodevelopmental disorder, we studied postnatal day seven (P7), 21 (P21) and adult P140 mice. MBP, a myelin protein responsible for myelin compaction in the central nervous system (CNS), was found to be significantly upregulated in corpus callosum of P7 *Shank3Δ11(−/−)* mice but downregulated in P21 and P140 both in immunostaining (Fig. 1c and Suppl. Figure 2c) and Western Blot analysis (Fig. 1d). MBP is expressed in four different isoforms, 14, 17, 18.5 and 21.5 kDa that appear as three distinct isoforms in the Western Blot. All of them were altered in the *Shank3Δ11(−/−)* mice. Furthermore, SHANK3 protein expression was analyzed during development at P7 and P21 (Suppl. Figure 2a). As in P140 mice, a reduction of the isoforms *a* and *c/d* was observed in *Shank3Δ11(−/−)*, whereas an increase of *300 kDa* and *e* was seen at P7. The number of intersections in the MBP staining can be used to closely analyze MBP expression, since more details are incorporated in the analysis compared to signal intensity and area [40]. Based on the number of intersections in the cortex, the complexity of MBP-positive processes was increased in P7 and decreased in P21 and P140 *Shank3Δ11(−/−)* mice (Fig. 1e). Western Blot analysis showed increased MBP expression in P7 and no changes at P21 and P140 (Suppl. Figure 2d). In striatum, MBP intensity was increased in P7, but no changes were observed in P21 and P140 animals (Fig. 1f) comparable to the Western Blot analysis (Suppl. Figure 2e). Nevertheless, the striatum displayed morphological changes of the white matter. The fibers of white matter in the striatum are called striosomes. These striosomes were smaller in *Shank3Δ11(−/−)* mouse brains (Fig. 1g). These results show impairment in white matter of *Shank3Δ11(−/−)* mice, both morphologically with a decreased volume in corpus callosum and smaller striosomes in striatum, and on a molecular level with alterations in MBP expression.

### SHANK3 localizes in myelinating cells

**Fig. 2:**
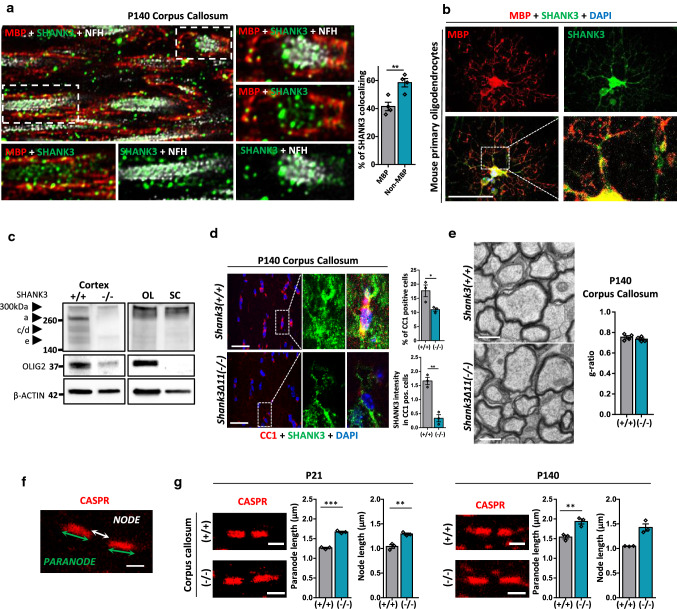
SHANK3 expression in myelinating cells. **a** SHANK3, MBP and NFH IHC in corpus callosum of 140-day-old *Shank3(*+ */* +*)* animals using expansion microscopy. The percentage of SHANK3 in the MBP compartment relative to the total SHANK3 content per fiber is shown. *n* = 4 animals. Mean ± SEM. Student’s Unpaired *t* test, *p* = 0.0073. **b** SHANK3 and MBP ICC in mouse primary oligodendrocytes. Scale bar 50 μm. **c** Western Blot analysis for SHANK3, OLIG2 and β-ACTIN in cortical lysates, primary oligodendrocytes (OL), and primary Schwann cells (SC). **d** SHANK3 and CC1 IHC and analysis in corpus callosum of 140-day-old *Shank3(*+ */* +*)* and *Shank3Δ11(−/−)* animals. Scale bar 15 μm. *n* = 3 animals. Mean ± SEM. CC1-positive: Student’s Unpaired *t* test, *p* = 0.0370. SHANK3/CC1-positive: Student’s Unpaired *t* test, *p* = 0.0017. **e** TEM analysis of corpus callosum of 140-day-old *Shank3(*+ */* +*)* and *Shank3Δ11(−/−)* animals. g-ratio was analyzed. Mean ± SEM. Scale bar 0.5 μm. *n* = 4 animals. Student’s Unpaired *t* test, *p* = 0.3674. **f** Paranodal CASPR immunofluorescence image. Measurement of paranode length (indicated by green arrow) and node length (indicated by white arrows). Scale bar 2 μm. **g** CASPR IHC and analysis in corpus callosum of 21 and 140 days old *Shank3(*+ */* +*)* and *Shank3Δ11(−/−)* animals. Scale bar 2 μm. *n* = 3 animals. Mean ± SEM. Student’s Unpaired *t* test, P21: paranode *p* = 0.0001, node *p* = 0.0038. P140: paranode *p* = 0.0074, node Mann–Whitney test *p* = 0.100

SHANK3 expression in cells of the oligodendrocyte lineage has not been reported up until now. Therefore, we co-stained for MBP and SHANK3 in corpus callosum of P140 mice and observed SHANK3 expression (Suppl. Figure 3a). The corpus callosum lacks dendrites and neuronal somas, suggesting that non-neuronal cells also express SHANK3. To exclude that the SHANK3 signal solely derived from axons, we performed expansion microscopy of P140 mouse brain sections. In the cortex (Suppl. Figure 3b) and in the corpus callosum, we found SHANK3 localizing in the axon, but also consistent SHANK3 expression in the MBP-positive myelin sheath surrounding the axon (Fig. 2a). The percentage of total SHANK3 content in the MBP-positive compartment per fiber was calculated (Suppl. Figure 3b) and approximately 40 to 55% of SHANK3 was found to localize there. O4-positive mouse primary oligodendrocytes (Suppl. Figure 3c) were stained for MBP and SHANK3 and a clear expression of SHANK3 in MBP-expressing cells was found (Fig. 2b). Furthermore, a Western Blot of oligodendrocyte, Schwann cell and mouse cortical lysate revealed clear SHANK3 expression (Fig. 2c). In addition, we analyzed CC1 expressed in the soma of oligodendrocytes. The number of CC1-positive cells was reduced in corpus callosum of P7, and significantly reduced in P21 (Suppl. Figure 3d) and P140 *Shank3Δ11(−/−)* mice (Fig. 2d, upper graph). This indicates not only a reduction of MBP expression but also a reduced number of oligodendrocytes in P21 and P140 *Shank3Δ11(−/−)* mice. CC1-positive cells expressed SHANK3 as well. Analysis of SHANK3 intensity in CC1-positive soma revealed residual but reduced SHANK3 expression in P140 *Shank3Δ11(−/−)* corpus callosum (Fig. 2d, lower graph). Ultrastructural analysis did not reveal changes in the g-ratio (Fig. 2e), which is calculated as the ratio between axon diameter and fiber diameter, or the axon diameter (Suppl. Figure 3e) in the corpus callosum of P140 mice. Also, analysis of 2′,3′-Cyclic-nucleotide 3′-phosphodiesterase (CNP), expressed in immature and mature oligodendrocytes, showed changes in P21 but not in P140 (Suppl. Figure 3f), suggesting maturation-dependent alterations.

To further investigate whether the SHANK3 and MBP loss in P21 and P140 mice might be associated with changes in the structure of the nodes of Ranvier, we immuno-stained for CASPR, a paranodal marker, and potassium channel 1.2 (Kv1.2), expressed in the juxta-paranodal zone (Fig. 2f and Suppl. Figure 4a–c). P7 has not been analyzed, since in this very early phase of myelination no signal was detectable. We observed an elongation of paranode (CASPR staining) and node length (in between two CASPR segments) in corpus callosum in P21 and P140, even though not significant in the P140 node (Fig. 2g), and in P21 and P140 cortex (Suppl. Figure 4a). In striatum, we observed the elongation only in the paranode of P140 *Shank3Δ11(−/−)* mice (Suppl. Figure 4b). In addition, the amount of Kv1.2-positive segments adjacent to CASPR was analyzed in P140 and a decrease was found in *Shank3Δ11(−/−)* in striatum and cortex (Suppl. Figure 4a–c). Thus, P21 and P140 *Shank3Δ11(−/−)* mice not only show a loss of SHANK3 and myelin proteins, but also changes in the node structure, suggesting possible alterations in signal propagation in these mice, since an increased node length has been associated with decreased axon conduction speed [41, 42].

### SHANK3 deficiency affects the myelin in the spinal cord

**Fig. 3:**
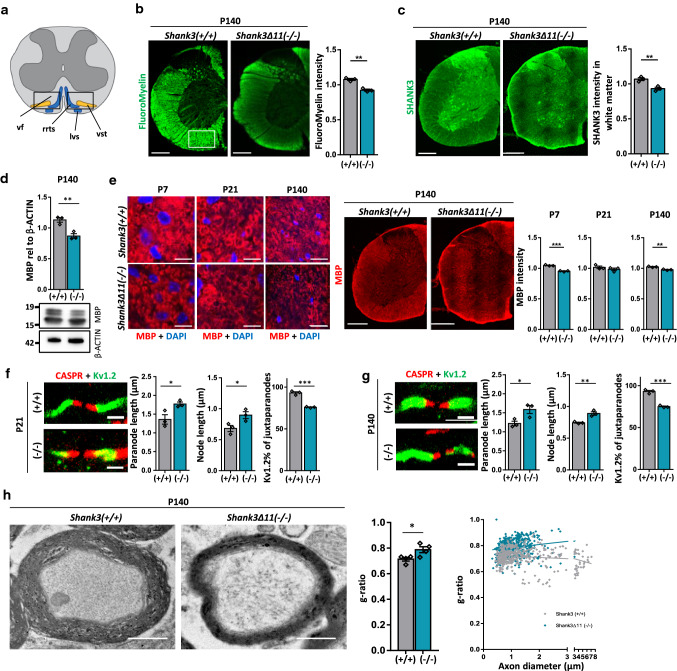
White matter and MBP expression are changed in the spinal cord of *Shank3Δ11(−/−)* mice. **a** Schematic illustration of the analyzed region in cervical spinal cord. vf: ventral funiculus, rrts: rostral reticulo-spinal tract, lvs: lateral vestibulo-spinal tract, vst: ventral spino-thalamic tract. Blue: descending fiber tract, yellow: ascending fiber tract. **b** FluoroMyelin intensity analysis in cervical spinal cord of 140-day-old *Shank3(*+ */* +*)* and *Shank3Δ11(−/−)* animals. Scale bar 500 μm. *n* = 3 animals. Mean ± SEM. Student’s Unpaired *t* test, *p* = 0.0016. **c** SHANK3 IHC and analysis in ventral cervical spinal cord of 140-day-old *Shank3(*+ */* +*)* and *Shank3Δ11(−/−)* animals. Scale bar 500 μm. *n* = 3 animals. Mean ± SEM. Student’s Unpaired *t* test, *p* = 0.0089. **d** Western Blot analysis of MBP and β-ACTIN in cervical spinal cord of 140-day-old *Shank3(*+ */* +*)* and *Shank3Δ11(−/−)* animals. *n* = 3 animals. Mean ± SEM. Student’s Unpaired *t* test, *p* = 0.0080. **e** MBP and DAPI IHC and analysis in ventral cervical spinal cord of 7-, 21- and 140-day-old *Shank3(*+ */* +*)* and *Shank3Δ11(−/−)* animals. Scale bar 20 μm. *n* = 3 animals. Mean ± SEM. Student’s Unpaired *t* test, P7 *p* = 0.0003, P21 *p* = 0.3205, P140 *p* = 0.0041. **f + g** CASPR and Kv1.2 IHC (labeled in red and green, respectively) and analysis in ventral lumbar spinal cord of 21- and 140-day-old *Shank3(*+ */* +*)* and *Shank3Δ11(−/−)* animals. Scale bar 2 μm. n = 3 animals. Mean ± SEM. Student’s Unpaired *t* test, P21: paranode *p* = 0.0388, node *p* = 0.0444, adjacent *p* = 0.0003. P140: paranode *p* = 0.0380, node *p* = 0.0063, adjacent *p* = 0.0003. **h** TEM analysis of ventral cervical spinal cord of 140-day-old *Shank3(*+ */* +*)* and *Shank3Δ11(−/−)* animals. *n* = 4 animals. Mean ± SEM. g-ratio was analyzed. Student’s Unpaired *t* test, *p* = 0.03. Scale bar 2 μm. g-ratio of individual axons as a function of axon diameter. *Shank3(*+ */* +*)* *R*^2^ = 0.0257, *Shank3Δ11(−/−)* R^2^ = 0.02904, Slope: *p* < 0.0001, Intercepts: S*hank3(*+ */* +*)* = 0.727, *Shank3Δ11(−/−)* = 0.7602

SHANK3 deficiency not only affects the brain but also all components of the voluntary motor system down to the muscle fiber [36] as well as the feedback from the periphery to the brain within the sensory system [43]. Therefore, we analyzed the ventral part of the cervical spinal cord that contains the ventral spino-thalamic tract that transmits touch and pressure perception back to the brain (Fig. 3a). We stained with the FluoroMyelin dye in the cervical part of the spinal cord, analyzing the ventral area of P140 *Shank3(*+ */* +*)* and *Shank3Δ11(−/−)* mice. FluoroMyelin intensity was decreased, among other areas, in the ventral white matter of *Shank3Δ11(−/−)* mice (Fig. 3b). Also, SHANK3 levels were found reduced in the white matter of the spinal cord in immunostaining (Fig. 3c) and in Western Blot analysis (Suppl. Figure 5a + b). We found that the SHANK3 isoforms in spinal cord gave a different isoform pattern when comparing to cortical lysate (Suppl. Figure 5c). However, the isoform pattern of spinal cord was similar to the sciatic nerve (Suppl. Figure 5d + e). MBP expression in the cervical spinal cord of P140 mice was significantly reduced in Western Blot analysis (Fig. 3d). Immunostainings revealed an MBP reduction in P7 and in P140 (Fig. 3e). Concerning SHANK3 expression in the spinal cord, we co-stained SHANK3 with NFH for the axonal and MBP for the myelin compartment. First, we found SHANK3 expression in both cell types, and second, we found reduced SHANK3 expression in *Shank3Δ11(−/−)* (Suppl. Figure 5f).

Furthermore, on longitudinal cervical sections, paranode and node length were assessed, and an elongation was observed at P21 (Fig. 3f) and P140 (Fig. 3g), in line with our findings in the brain. In addition, the number of Kv1.2 segments adjacent to CASPR was significantly reduced both in P21 and P140 *Shank3Δ11(−/−)* mice (Fig. 3f + g, right graph). Using electron microscopy, we observed a significant increase of the g-ratio in P140 *Shank3Δ11(−/−)* mice (Fig. 3h). This indicates reduced myelin thickness, since no changes in axon diameters were detected (Suppl. Figure 5g), consistent with our finding of less MBP expression.

### SHANK3 deficiency alters the myelin in the peripheral nervous system (PNS)

**Fig. 4:**
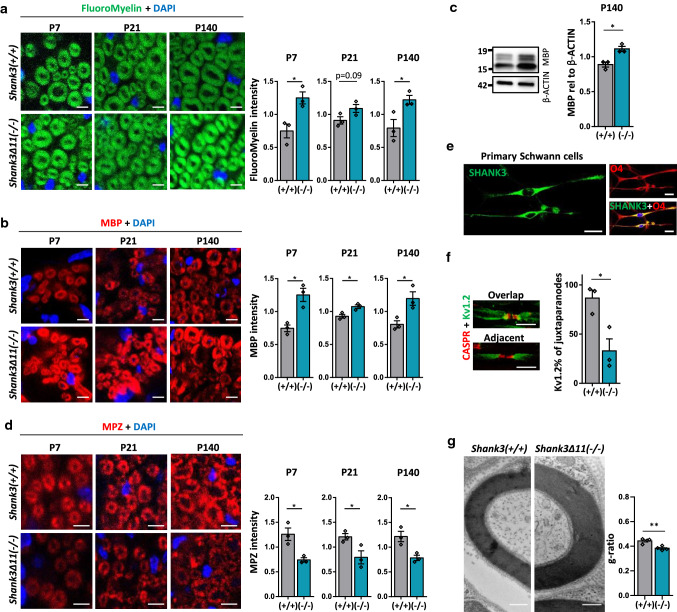
White matter, MBP and MPZ expression are changed in the sciatic nerve of *Shank3Δ11(−/−)* mice. **a** FluoroMyelin analysis and DAPI staining in sciatic nerve of 7-, 21- and 140-day-old *Shank3(*+ */* +*)* and *Shank3Δ11(−/−)* animals. Scale bar 5 µm. *n* = 3 animals. Mean ± SEM. Student’s Unpaired *t* test, P7 *p* = 0.0224, P21 *p* = 0.0912, P140 *p* = 0.0425. **b** MBP and DAPI IHC and analysis in sciatic nerve of 7-, 21- and 140-day-old *Shank3(*+ */* +*)* and *Shank3Δ11(−/−)* animals. Scale bar 5 µm. *n* = 3 animals. Mean ± SEM. Student’s Unpaired *t* test. P7 *p* = 0.0104, P21 *p* = 0.0205, P140 *p* = 0.0280. **c** Western Blot analysis of MBP and β-ACTIN in sciatic nerve of 140-day-old *Shank3(*+ */* +*)* and *Shank3Δ11(−/−)* animals. *n* = 3 animals. Mean ± SEM. Student’s Unpaired *t* test, *p* = 0.0117. **d** MPZ and DAPI IHC and analysis in sciatic nerve of 7-, 21- and 140-day-old *Shank3(*+ */* +*)* and *Shank3Δ11(−/−)* animals. Scale bar 10 µm. *n* = 3 animals. Mean ± SEM. Student’s Unpaired *t* test. P7 *p* = 0.0177, P21 *p* = 0.0477, P140 *p* = 0.0205. **e** SHANK3 localization in O4-positive primary Schwann cells. Scale bar 20 µm. **f** CASPR and Kv1.2 IHC in sciatic nerve of 140-day-old *Shank3(*+ */* +*)* and *Shank3Δ11(−/−)* animals. Scale bar 10 µm. n = 3 animals. Percentage of adjacent CASPR and Kv1.2 structures. Mean ± SEM. Student’s Unpaired *t* test, *p* = 0.0216. **g** TEM analysis of sciatic nerve. Myelinated axons of 140-day-old *Shank3(*+ */* +*)* and *Shank3Δ11(−/−)* animals and g-ratio analysis. Student’s Unpaired *t* test, *p* = 0.0035

As SHANK3 deficiency leads to alterations in the neuromuscular junction and SHANK3 was also expressed in Schwann cells, the myelinating cells of the peripheral nervous system (PNS), we analyzed the sciatic nerve to study possible effects of SHANK3 deficiency. We evaluated the FluoroMyelin intensity in sciatic nerves of P7, P21 and P140 mice and observed an increase in *Shank3Δ11(−/−)* mice (Fig. 4a). Along with that, increased expression of MBP was found in P7, P21 and P140 mice (Fig. 4b), also confirmed by Western Blot analysis of P140 mice (Fig. 4c). In contrast to the oligodendrocytes in the CNS, we saw increased myelin and MBP expression in all ages of *Shank3Δ11(−/−)* mice in the PNS. We also analyzed Myelin Protein Zero (MPZ), an important myelin protein in the PNS [44]. We found decreased MPZ expression in all ages of *Shank3Δ11(−/−)* (Fig. 4d), similar to the P21 and P140 findings of MBP in the CNS.

SHANK3 expression was reduced in sciatic nerve protein lysate (Suppl. Figure 5d + e). We analyzed SHANK3 expression in sciatic nerve at P7, P21 and P140 in both the MBP-positive myelinating cells and the NFH-positive axonal compartments and found, again, a localization of SHANK3 in both compartments and reduced SHANK3 expression in *Shank3Δ11(−/−)* mice (Suppl. Figure 6a), linking the myelin changes to SHANK3 deficiency. To confirm these findings on a cellular level, mouse primary Schwann cells were cultured. SHANK3 localized in these cells in immunostaining (Fig. 4e) and SHANK3-specific isoforms were detected in Western Blot (Fig. 2c). We also analyzed paranodal and nodal structures by CASPR and Kv1.2 immunostaining in the PNS. No changes in nodal and paranodal length were seen in *Shank3Δ11(−/−)* mice in P21 and P140 mice (Suppl. Figure 6b). However, a significantly lower number of Kv1.2-positive structures was observed to localize with CASPR segments or being adjacent to them (Fig. 4f). To form a functional unit in the nodes of Ranvier, CASPR and Kv1.2 need to be observed in close proximity, overlapping or adjacent. Our findings suggest a possible reduction in axon conduction speed also in the PNS of *Shank3Δ11(−/−)* mice [41]. Furthermore, we used electron microscopy to examine the sciatic nerves of *Shank3(*+ */* +*)* and *Shank3Δ11(−/−)* mice. The axon diameter remained unchanged (Suppl. Figure 6c), but the g-ratio was decreased (Fig. 4g) pointing toward thicker myelin in the PNS of *Shank3Δ11(−/−)* mice. In summary, in the PNS we observed increased MBP expression, but decreased MPZ in *Shank3Δ11(−/−)* mice. However, the thickness of the myelin was increased.

### Number and intensity of MBP-expressing cells is reduced in hiPSC-derived cerebral organoids of PMDS patients

**Fig. 5:**
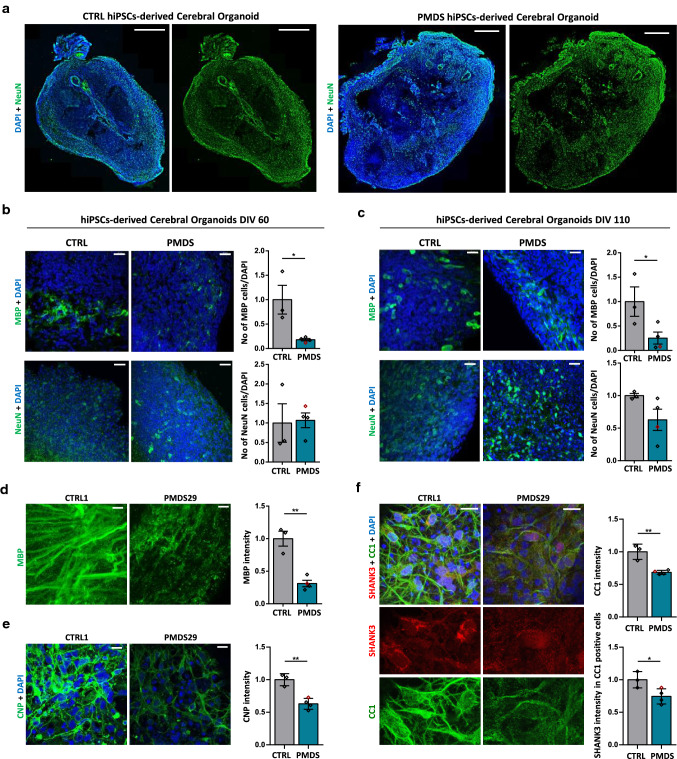
Human cerebral organoids recapitulate the MBP changes seen in vivo. **a** IHC for NeuN and DAPI in DIV 110 hiPSC-organoids of CTRL and PMDS. Scale bar 500 μm. **b** IHC for MBP and DAPI, NeuN and DAPI in DIV 60 hiPSC-organoids of CTRL and PMDS. The InsG line is indicated in red. Number of MBP-positive cells: Student’s Unpaired *t* test, *p* = 0.0212. Mean ± SEM. Number of NeuN positive cells: Student’s Unpaired *t* test, *p* = 0.8895. Scale bar 30 μm.** c** IHC for MBP and DAPI, NeuN and DAPI in DIV 110 hiPSC-organoids of CTRL and PMDS. Number of MBP-positive cells: Student’s Unpaired *t* test, *p* = 0.0496. Mean ± SEM. Number of NeuN positive cells: Student’s Unpaired *t* test, *p* = 0.1156. Scale bar 30 μm. **d** IHC for MBP and DAPI in DIV 110 hiPSC-organoids of CTRL and PMDS. The InsG line is indicated in red. *n* = 3 organoids per cell line. Mean ± SEM. MBP intensity: Student’s Unpaired *t* test, *p* = 0.0017. Scale bar 30 μm**. e** CNP IHC in DIV120 hiPSC-derived air–liquid interface cerebral organoids (ALI-COs) of CTRL and PMDS. The InsG line is indicated in red. Student’s Unpaired *t* test, *p* = 0.0026. Scale bar 10 μm. **f** SHANK3 and CC1 IHC in DIV120 hiPSC-derived ALI-COs of CTRL and PMDS**.** The InsG line is indicated in red. Mean ± SEM. Student’s Unpaired *t* test. CC1 intensity *p* = 0.0032. SHANK3 in CC1-positive *p* = 0.0382. Scale bar 10 μm

To investigate whether our findings in mice also hold true for human tissue, young DIV60 and mature DIV110 cerebral organoids were analyzed. We generated cerebral organoids of three control and three Phelan–McDermid syndrome (PMDS) patient hiPSC lines that have a heterozygous deletion of *SHANK3*. In addition, we included a CRISPR/Cas9 engineered line (InsG) that, based on CTRL1, harbors an inserted guanine leading to a premature STOP codon in *SHANK3* (published in [36]) to ensure SHANK3 specificity of our results. The PMDS and the InsG lines are shown together as PMDS. At DIV 110, CTRL and PMDS organoids displayed a mature morphology with compartmentalization, expressing NeuN (Fig. 5a). In the cortex-like border zone, the number of MBP-expressing cells was reduced both at DIV 60 (Fig. 5b) and 110 (Fig. 5c), while the number of NeuN positive neurons was the same in CTRL and PMDS organoids (Fig. 5b, c). Furthermore, a reduction of MBP expression was found in PMDS SHANK3-deficient organoids at DIV110 (Fig. 5d), comparable to our results in mice. This indicates that the reduction of MBP expression under SHANK3 deficiency is not a mouse-specific phenomenon but also presents in cells of human origin.

Further following the changes observed in *Shank3Δ11(−/−)* mice in myelinating cells, organoids were stained for CNP, and a reduced expression was found in PMDS (Fig. 5e), suggesting again that oligodendrocytes are affected by SHANK3 loss. In addition, CC1 expression showed a decrease in PMDS organoids (Fig. 5f). Comparable to the data obtained in mouse tissue, SHANK3 was expressed in these CC1-positive cells, and SHANK3 intensity was reduced in PMDS organoids (Fig. 5f, lower graph). In summary, the data obtained from hiPSC-derived organoids strongly support our findings in mouse tissue, increasing the relevance of our findings for patients and offering possibilities for future drug screening in human-derived material.

## Discussion

This study is based on previous findings of MRI analysis in PMDS patients [30, 31] and *Shank3Δ11(−/−)* mice [29] with profound loss of white matter integrity in SHANK3 deficiency. We used *Shank3Δ11(−/−)* mice and hiPSC-derived organoids of PMDS patients and have, to our knowledge, performed the first study analyzing molecular changes in white matter under SHANK3 deficiency.

Myelination, the process of myelin formation, is crucial for the conduction velocity in fiber tracts in the nervous system and is necessary for the proper transmission of electrical signals in neuronal networks. Fiber tracts of the white matter link different brain regions enabling the control of cognition and behavior [12]. Alterations of the white matter are a recurrent finding in ASD pathology (reviewed in [45]) and often referred to as disturbance of connectivity. More than a decade ago, a local hyper-connectivity within brain regions and a long-distance hypo-connectivity has been postulated [46]. The structure of the white matter has been analyzed in ASD children and matched controls and alterations have been observed in cortical regions important for social cognition and the integration of information [47]. The first three years of life are the most dynamic period of myelination in the human brain. The myelin sheath is built, and axons are enwrapped [48]. Analysis of the corpus callosum, the major inter-hemispheric white matter tract, revealed a significantly increased area and thickness in the majority of children with ASD under 2 years of age [49]. Therefore, corpus callosum overgrowth has even been postulated to be among the earliest neural signatures of ASD [49]. In this respect, the increased expression of MBP we observed in P7 *Shank3Δ11(−/−)* mice could well resemble such accelerated myelination. The decreased volume of the corpus callosum we found in adult *Shank3Δ11(−/−)* mice has also been described in SHANK3-deficient macaques. These showed alterations in local and global connectivity patterns in MRI, indicative for circuit abnormalities [50]. In addition, Shank3-deficient rats presented a reduced brain volume and impairments in white matter tracts [51]. These data support the hypothesis, that SHANK3 deficiency is another disorder of the autistic spectrum where the investigation of white matter changes and myelin might be essential to fully understand the disorder and its underlying mechanisms.

In this study, we showed the expression of SHANK3 in oligodendrocytes and Schwann cells and, therefore, we suggest a cell autonomous effect in those cells that eventually leads to the profound alterations in myelin we observed in SHANK3 deficiency. These observations indicate that the role of SHANK3 in neural tissue has been underestimated so far, since mainly the gray but not the white matter has been studied. The alterations of myelin under SHANK3 deficiency described in this study provides good evidence to further develop our understanding of SHANK3 in myelination. To date, PMDS has mainly been seen as a synaptopathy, but should eventually be considered as a combination of synaptopathy and myelinopathy. Of note, we did not observe any changes in axon diameter in any of the tissues analyzed. Nevertheless, it has been reported that axons are affected by SHANK3 loss. Neurite arborization was diminished in SHANK3-deficient primary hippocampal neurons [52], and also the neurite length and the growth cones area were smaller compared to wild type conditions [53]. Thus, it still has to be determined, how the loss of SHANK3 in the different neural cell types play together and contribute to the ultimate phenotype of alterations in both gray and white matter of SHANK3-deficient subject [54]. Putting SHANK3 deficiency at the interplay of being a synaptopathy or a myelinopathy, one could argue that the disturbance of myelin might lead to a disturbance of neuronal networks and neuronal activity, as already suggested by Jesse et al. 2019 [30]. In this respect, further research can provide insight into what sequence of developmental events results in the observed alterations.

The putative role of SHANK3 in myelination is still unknown, however, myelin-associated changes have repeatedly been reported by the group of Han [55–57]. Performing gene ontology analysis in a brain region-specific interactome of SHANK3, a significant enrichment in myelin sheath-associated genes has been reported in all regions analyzed (medial prefrontal cortex, hippocampus, and striatum) [56]. Furthermore, the group analyzed the transcriptome of a SHANK3 overexpressing mouse in the striatum and the interactome network also contained an MBP-SHANK3 interaction [57]. In addition, this SHANK3 overexpressing mouse model has shown decreased mRNA levels of myelin-related genes in the medial prefrontal cortex, manifesting significant changes in the gene sets, such as “myelination” and “myelin sheath” [55]. On the level of individual genes, MBP was found to be decreased [55]. These findings support our observations and strengthen the connection between SHANK3 dosage and a disturbance of myelin composition including MBP expression.

ASD is considered a neurodevelopmental disorder, meaning that neural alterations observed in patients might mainly derive from disturbances during development. Our organoid data revealed changes in the number of myelinating cells expressing MBP, CC1 and CNP already at an early age during neurodevelopment. In our hand, these organoids did not show myelination of axonal fibers yet, but the presence of oligodendrocyte progenitor cells (OPCs) has been shown before [58]. In our study, cerebral organoids not especially enriched in oligodendrocytes have been generated [33], since they offer the advantage of a growth factor driven differentiation of a physiological mixture of neural cells allowing the close observation of development and maturation of cells. Our data indicate a lack and/or a prolongation of maturation in PMDS organoids, and help understanding the neurodevelopmental perspective of SHANK3-ASD. Taken together with the mouse data, we observe a defect in maturation of oligodendrocytes, an increase in myelin and MBP at an early postnatal age and lower levels of myelin together with an altered myelin composition in adult animals. The increased MBP expression at P7 in *Shank3Δ11(−/−)* mice in most regions analyzed (corpus callosum, cortex, striatum, and the peripheral nerve) is especially interesting. Speculating about possible reasons for the increased MBP expression at P7, one possibility would be that the lack of SHANK3 in its role as a scaffolding protein, both in precursors for neurons and oligodendrocytes, disturbs the development of the timed pattern of expression of downstream proteins and one of them might be MBP. At P7, a peak of oligodendrocyte proliferation is reached during development in mice [59], that goes along with an approximate 50% overproduction of oligodendrocytes [60]. These excess cells are dying after failing to make contact to an axon. Within the very dynamic environment of this peak, the system might either ‘over-react’ leading to increased MBP levels or not be able to get rid of these excess oligodendrocytes in time. Once this peak is overcome, the system might try to reach a steady state that then results in decreased MBP expression due to the loss of SHANK3.

In other ASD models, MBP-associated changes have already been reported. A dynamic expression of MBP during neurodevelopment that is normalized in adult mice, has been reported in several ASD mouse models. P7 mice modeling Fragile X Syndrome demonstrated a reduced volume of the cerebellum together with an 80% reduction of MBP expression [16]. However, at P30, normal MBP expression was seen [16]. The authors describe a dynamic process in an ASD model, where developmental hypomyelination results in normal myelination in adult mice. Contrarily, in BTBR mice, a mouse model of idiopathic ASD, increased volumes of fiber tracts have been observed at P6, along with increased MBP expression. In adult P35 mice, MBP expression was again normal compared to control [15]. This precocious myelination of the BTBR mouse is comparable to our observations in the *Shank3Δ11(−/−)* mice at P7. Since it is not known whether this increased myelination also occurs in SHANK3-deficient PMDS patients, it would be of interest to investigate brain and corpus callosum size in PMDS patients under the age of two. To date, MRI studies have been performed in small cohorts of patients with PMDS. Soorya et al. have revealed changes in corpus callosum in approximately 50% of the PMDS patients investigated that included thinning or hypoplasia, white matter changes, delayed myelination and generalized white matter atrophy [61]. Thinning of the corpus callosum in patients with PMDS has been reported by further publications [62–64]. The dynamic dysregulation of MBP expression we observed in the *Shank3Δ11(−/−)* mice might give a possible explanation for these myelin alterations in PMDS. Furthermore, it reveals that the common finding of atypical myelination in ASD has distinct and jointed traits in regard to the underlying etiology. However, longitudinal studies investigating white matter alterations in PMDS patients are needed to understand the dynamics of pathology progression.

Besides increased myelin and MBP at P7, we found decreased myelin levels in corpus callosum, cortex and spinal cord in adult animals. In spinal cord, the thickness of the myelin sheath was decreased on an ultrastructural level accordingly, while in corpus callosum such alterations were not visible. Alterations of MBP expression usually are reported to be accompanied by changes in myelin thickness and g-ratio, respectively [65, 66]. MBP is an essential structural protein of the myelin sheath ensuring the compaction of the myelin membranes [67]. Therefore, we would have expected a change in the g ratio as well. However, the loss of MBP might also be compensated on the *Shank3Δ11(−/−)* mice by other myelin proteins, that have not been included in this study.

While the increased MBP content we observed at P7 could explain the delayed achievement of developmental milestones in PMDS, that include motor function, cognitive abilities, and social achievements [68, 69], the decrease of myelination and MBP observed at P21 and P140, might explain the regression observed in patients with PMDS. Regression in PMDS most frequently has an onset in mid-childhood and affects motor and self-help skills [70, 71]. In addition, we also observed structural changes of the nodes of Ranvier, depicted by an elongated paranode and node length, in adult *Shank3Δ11(−/−)* mice. Mature oligodendrocytes maintain the capacity to adjust paranode length and node elongation is a pathological phenomenon associated with myelin loss or paranodal changes (summarized in [72]). These alterations suggest a changed signal propagation and axonal conductance in the *Shank3Δ11(−/−)* mice, that might also reflect in regression of PMDS patients. Therefore, the loss of MBP might directly impact on the lengths of node and paranode.

In contrast to the CNS, we observed increased myelin and MBP expression together with decreased myelin protein zero (MPZ) expression in the PNS at all ages analyzed. In adult P140 mice, the thickness of the myelin sheath was increased. Myelination in the PNS is arranged fundamentally differently from the CNS, even though the resulting myelin and enwrapping of axons are ultra-structurally comparable. Schwann cells derive from the embryonic neural crest [73], and each Schwann cell myelinates only one separate segment along an axon, unlike the oligodendrocytes in the CNS that enwrap multiple segments of different axons [74]. The Schwann cell physiology differs from oligodendrocytes, for example, they can degrade myelin, de-differentiate, divide, re-myelinate and regenerate (reviewed in [73]). Furthermore, they differ in the protein composition of the compact myelin. While the CNS contains MBP and proteolipid protein (PLP) as dominant myelin proteins, the role of MBP is taken by MPZ in the PNS to adhere two adjacent myelin membranes together leading to myelin compaction [75]. In the CNS, mice harboring the so called Shiverer mutation in the MBP gene manifest with a myelin malformation [76]. In the PNS, those mice lose MBP as well, but the myelin remains compact. The authors explain this occurrence by the expression of MPZ that is not changed upon MBP loss [77]. In the CNS and PNS, a positive correlation between MBP expression and the myelin thickness has been reported [78]. The increased FluoroMyelin intensity we observed in the sciatic nerve of *Shank3Δ11(−/−)* mice, might be driven by the increased MBP expression. Since MPZ is expressed prior to MBP, an MPZ deficiency may be over-compensated by later synthesized myelin proteins such as MBP [79] leading to an increased myelin sheath thickness in adult mice. Interestingly, we found in the sciatic nerve less CASPR-positive structures to be correctly localized next to a Kv1.2 segment. The paranode, visualized by the CASPR staining, acts as a barrier to keep the nodal proteins apart from the Kv1.2 channels in the juxta-paranode. An increase of node length has been observed in multiple neurological disorders (reviewed in [80]). This lengthening might occur due to myelin retraction and cause changes in current flows under the myelin. However, the mechanisms underlying these changes have not yet been determined.

We found SHANK3 expression in both oligodendrocytes and Schwann cells. However, the expressed SHANK3 isoforms seem to be different, suggesting that the expression of distinct SHANK3 isoforms might affect the expression of MBP differentially, leading to a decrease in the CNS and an increase in the PNS. Ultimately, SHANK3 deficiency leads to loss of MBP in the CNS and to loss of MPZ in the PNS, both being important myelin proteins of the respective tissues. So far, we can only speculate how the loss of SHANK3 indeed might lead to deficits in myelination, differentiation and function of oligodendrocytes. Given the known scaffolding role of SHANK3 in neurons, SHANK3 might function similarly in myelinating cells and a loss of stability might contribute to the alterations we observed. SHANK3 might anchor receptor molecules that then get lost under SHANK3 deficiency. The resulting lack could then result in the observed changes in myelination and the expression of MBP. In respect of development, the interplay between neurons and myelinating cells is essential for the proper formation of connections. One could speculate that the loss of SHANK3 in neurons also impacts on the differentiation of oligodendrocytes and Schwann cells, demanding the analysis of the interaction of the two cell types in future experiments.

In respect of patients with PMDS, a variety of comorbidities can be attributed to those myelin-related changes we observed. For example, SHANK3-deficient patients manifest with a decreased perception of pain [68, 69]. The increased thickness of the myelin sheath in adult *Shank3Δ11(−/−)* mice in the PNS might hinder the accurate signal transmission from the periphery to CNS. Furthermore, it might explain the general delay in development of patients with PMDS, assuming that signal integration of sensory information from the periphery is essential for proper formation of circuits in the central nervous system. MRI studies showed that both commissure and projection fibers are affected in PMDS [30]. The possible developmental mis-projection of fibers might explain multiple phenotypes observed in PMDS, that so far have been attributed to underlying synaptic disturbances.

## Supplementary Information

Below is the link to the electronic supplementary material.

**Supplementary Figure 1. a** MRI analysis of corpus callosum volume in 4-week-old *Shank3(+/+)* and *Shank3Δ11(−/−) *animals. Males: n=10 +/+, n=5 −/− animals. Females: n=7 +/+, n=4 −/− animals Mean±SEM. Student’s Unpaired t-test, total p=0.0075, right p=0.0153, left p=0.0048, total males p=0.1523, right males p=0.2914, left males p=0.0892, total females p=0.0151, right females p=0.0106, left females p=0.0212. **b** MRI analysis of corpus callosum volume in 9-week-old *Shank3(+/+)* and *Shank3Δ11(−/−) *animals. n=10 +/+, n=5 −/− animals. Mean±SEM. Student’s Unpaired t-test, total p=0.0009, right p=0.0020, left p=0.0006, total males p=0.0211, right males p=0.0333, left males p=0.0149, total females p=0.0132, right females p=0.0206, left females p=0.0119. **c** Total brain volume analysis in 4-week-old *Shank3(+/+)* and *Shank3Δ11(−/−) *animals. n=10 +/+, n=5 −/− animals. Mean±SEM. Student’s Unpaired t-test, total p=0.0156, males p=0.1843, females p=0.0046. Total brain volume analysis in 9-week-old *Shank3(+/+)* and *Shank3Δ11(−/−) *animals. n=10 +/+, n=5 −/− animals. Mean±SEM. Student’s Unpaired t-test, total p=0.2736, males p=0.5036, females p=0.2798. **d **MBP IHC and analysis of gray and white matter thickness of 140-day-old *Shank3(+/+)* and *Shank3Δ11(−/−) *animals. Scale bar 500μm. n=3 animals. Mean±SEM. Student’s Unpaired t-test. Corpus callosum: distance was measured from the mid-line up to 1200μm. Mid-line: p=0.0226, 200μm p=0.0222, 400μm p=0.0033, 600μm p=0.0098, 800μm p=0.0272, 1000μm p=0.2496. 1200μm p=0.4404. Cortex: 200μm p=0.6818, 400μm p=0.5539, 600μm p=0.2489, 800μm p=0.0953, 1000μm p=0.3735, 1200μm p=0.2858. **Supplementary Figure 2. a** Western Blot analysis of SHANK3 and β-ACTIN in corpus callosum of 7-, 21- and 140-day-old *Shank3(+/+)* and *Shank3Δ11(−/−) *animals. n=3 animals. Mean±SEM. Student’s Unpaired t-test. P7: 300kDa p=0.0628, c/d p=0.0011, e p<0.0001. Mann-Whitney test. P7: a p=0.1000 P21: 300kDa p=0.8707, a p=0.00734, c/d p=0.0009, e p=0.1764; P140: 300kDa p=0.0831, a p=0.0044, c/d p=0.0011, e p=0.0254. **b **FluoroMyelin intensity analysis in cortex, striatum of 140-day-old *Shank3(+/+)* and *Shank3Δ11(−/−) *animals. n=4 animals. Mean±SEM. Student’s Unpaired t-test, cortex: p=0.0005, striatum: p=0.3498. **c** MBP IHC in corpus callosum, cortex and striatum of 7-, 21- and 140-day-old *Shank3(+/+)* and *Shank3Δ11(−/−) *animals. Scale bar 30μm. **d** Western Blot analysis of MBP and β-ACTIN in cortex of 7-, 21- and 140-day-old *Shank3(+/+)* and *Shank3Δ11(−/−) *animals. n=3 animals. Mean±SEM. Student’s Unpaired t-test. P7 p=0.0009, P21 p=0.6847. P140 p=0.0584. **e** Western Blot analysis of MBP and β-ACTIN in striatum of 7-, 21- and 140-day-old *Shank3(+/+)* and *Shank3Δ11(−/−) *animals. n=3 animals. Mean±SEM. Student’s Unpaired t-test, P7 p<0.0001, P21 p=0.4945, P140 p=0.8008. **Supplementary Figure 3. a** SHANK3 and MBP IHC in corpus callosum of 140-day-old *Shank3(+/+) *animals. Scale bar 50μm. **b** SHANK3, MBP and NFH IHC in cortex of 140-day-old *Shank3(+/+) *animals using expansion microscopy. The percentage of SHANK3 in the MBP compartment relative to the total SHANK3 content per fiber is shown. Scale bar 10μm. n=4 animals. Mean±SEM. Student’s Unpaired t-test, p=0.0480. **c** ICC for O4 and DAPI in primary oligodendrocytes. **d **SHANK3 and CC1 IHC and analysis in corpus callosum of 7- and 21-day-old *Shank3(+/+)* and *Shank3Δ11(−/−) *animals. Scale bar 15μm. n=3 animals. Mean±SEM. P7 Mann–Whitney test CC1^+^ p=0.100, P21 Student’s Unpaired t-test CC1^+^ p=0.0082. **e **TEM analysis of corpus callosum of 140-day-old *Shank3(+/+)* and *Shank3Δ11(−/−) *animals. g-ratio of individual axons as a function of axon diameter. *Shank3(+/+)* R²=0.0257, *Shank3Δ11(−/−) *R²=0.02904, Slope: p<0.0001, Intercepts: S*hank3(+/+)* = 0.727, *Shank3Δ11(−/−) *= 0.7602. Axonal diameters were measured. n=4 animals. Mean±SEM. Student’s Unpaired t-test, p=0.2493. **f** CNP and DAPI IHC and analysis in corpus callosum, cortex and striatum of 21- and 140-day-old *Shank3(+/+)* and *Shank3Δ11(−/−) *animals. Scale bar 30μm. n=3 animals. Mean±SEM. Corpus callosum P21 Student’s Unpaired t-test p=0.0581. P140 Mann–Whitney test p=0.100. Cortex P21 Student’s Unpaired t-test p=0.0467, P140 Student’s Unpaired t-test p=0.7333. Striatum P21 Student’s Unpaired t-test p=0.0153, P140 Mann–Whitney test p=0.1000. **Supplementary Figure 4. a** CASPR and Kv1.2 IHC and analysis in cortex of 21- and 140-day-old *Shank3(+/+)* and *Shank3Δ11(−/−) *animals. Scale bar 2μm. n=3 animals. Mean±SEM. Student’s Unpaired t-test, P21 paranode p=0.0372, node p=0.4507. P140 paranode p=0.0252, node p=0.0107, adjacent p=0.0634. **b** CASPR and Kv1.2 IHC and analysis in striatum of 21- and 140-day-old *Shank3(+/+)* and *Shank3Δ11(−/−) *animals. Scale bar 2μm. n=3 animals. Mean±SEM. Student’s Unpaired t-test, P21 paranode p=0.6024, node p=0.2235, P140 paranode p=0.0219, node p=0.0112, adjacent p=0.0179. **c **CASPR and Kv1.2 IHC and analysis in corpus callosum of 140-day-old *Shank3(+/+)* and *Shank3Δ11(−/−) *animals. Scale bar 2μm. n=3 animals. Mean±SEM. Student’s Unpaired t-test. Adjacent p=0.9695. **Supplementary Figure 5. a+b** Western Blot analysis of SHANK3 and β-ACTIN in cervical spinal cord of 140-day-old *Shank3(+/+)* and *Shank3Δ11(−/−) *animals. n=3 animals. Mean±SEM. Student’s Unpaired t-test, 300kDa p=0.0719, a p=0.0559, e p=0.0587, f p=0.0002. Mann–Whitney test, e_1_ p>0.9999. **C **Western Blot of SHANK3 and β-ACTIN of cortex of 140-day-old *Shank3(+/+)* animals. **d+e** Western Blot analysis of SHANK3 and β-ACTIN in sciatic nerve of 140-day-old *Shank3(+/+)* and *Shank3Δ11(−/−) *animals. n=3 animals. Mean±SEM. Student’s Unpaired t-test, 300kDa p=0.0625, a p=0.0149, e p=0.0086, e_1_ p=0.1420, f p=0.0428. **f** SHANK3, MBP, NFH and DAPI IHC and analysis in cervical spinal cord of 7-, 21- and 140-day-old *Shank3(+/+)* and *Shank3Δ11(−/−) *animals. Scale bar 10μm. n=3 animals. Mean±SEM. Student’s Unpaired t-test. P7 SHANK3 intensity p=0.0014, SHANK3/MBP p=0.0004, SHANK3/NFH p=0.0034. P21 SHANK3 intensity p=0.0113, SHANK3/MBP p=0.0607, SHANK3/NFH p=0.0050. P140 SHANK3/MBP p=0.0104, SHANK3/NFH p=0.0183. **g **TEM analysis of ventral cervical spinal cord of 140-day-old *Shank3(+/+)* and *Shank3Δ11(−/−) *animals. n=4 animals. Mean±SEM. Axonal diameters were measured. Student’s Unpaired t-test, p=0.3572. **Supplementary Figure 6. a** SHANK3, MBP, NFH and DAPI IHC and analysis in sciatic nerve of 7-, 21- and 140-day-old *Shank3(+/+)* and *Shank3Δ11(−/−) *animals. Scale bar 5µm. n=3 animals. Mean±SEM. P7 SHANK3 Mann–Whitney test p=0.0100. SHANK3/MBP Student’s Unpaired t-test p=0.0229, SHANK3/NFH Student’s Unpaired t-test p=0.2390. P21 SHANK3 Mann–Whitney test p=0.0100. SHANK3/MBP Mann–Whitney test p=0.4000, SHANK3/NFH Student’s Unpaired t-test p=0.2362. P140 SHANK3 Student’s Unpaired t-test p=0.0476. SHANK3/MBP Student’s Unpaired t-test p=0.0471, SHANK3/NFH Student’s Unpaired t-test p=0.3827. **b **CASPR IHC and analysis in sciatic nerve of 21- and 140-day-old *Shank3(+/+)* and *Shank3Δ11(−/−) *animals. Scale bar 2µm. n=3 animals. Mean±SEM. Student’s Unpaired t-test. P21 paranode p=0.1410, node p=0.3180. P140 paranode p=0.3552, node p=0.2560. **c** TEM analysis of sciatic nerve of 140-day-old *Shank3(+/+)* and *Shank3Δ11(−/−) *animals. Axon diameter was measured. Scale bar 5μm. n=4 animals. Mean±SEM. Student’s Unpaired t-test, p=0.9020. The correlation between g-ratio and axonal diameter is showed. *Shank3(+/+)* R²=0.4007, *Shank3Δ11(−/−) *R²=0.3462, Slope: p=0.6381, Intercepts: p<0.0001. (PDF 2062 KB)

## Declarations

### Data availability

The datasets generated during and/or analyzed during the current study are available from the corresponding author on reasonable request.

## References

[1] DSM-V DDMNSYPICfDCP,  (2009). Prevalence of autism spectrum disorders—Autism and Developmental Disabilities Monitoring Network, United States, 2006. Morb Mortal Wkly Rep Recomm Rep.

[2] Developmental Disabilities Monitoring Network Surveillance Year Principal I, Centers for Disease C, Prevention. Prevalence of autism spectrum disorder among children aged 8 years - autism and developmental disabilities monitoring network, 11 sites, United States, 2010. Morbidity and mortality weekly report Surveillance summaries. 2014;63(2):1–21. 24670961

[3] Grabrucker AM (2012). Environmental factors in autism. Front Psych.

[4] Modabbernia A, Velthorst E, Reichenberg A (2017). Environmental risk factors for autism: an evidence-based review of systematic reviews and meta-analyses. Mol Autism.

[5] Zoghbi HY, Bear MF (2012). Synaptic dysfunction in neurodevelopmental disorders associated with autism and intellectual disabilities. Cold Spring Harbor Perspect Biol..

[6] Ismail MM, Keynton RS, Mostapha MM, ElTanboly AH, Casanova MF, Gimel'farb GL (2016). Studying autism spectrum disorder with structural and diffusion magnetic resonance imaging: a survey. Front Hum Neurosci.

[7] Keller TA, Kana RK, Just MA (2007). A developmental study of the structural integrity of white matter in autism. NeuroReport.

[8] Sparks BF, Friedman SD, Shaw DW, Aylward EH, Echelard D, Artru AA (2002). Brain structural abnormalities in young children with autism spectrum disorder. Neurology.

[9] Courchesne E, Karns CM, Davis HR, Ziccardi R, Carper RA, Tigue ZD (2001). Unusual brain growth patterns in early life in patients with autistic disorder: an MRI study. Neurology.

[10] Redcay E, Courchesne E (2005). When is the brain enlarged in autism? A meta-analysis of all brain size reports. Biol Psychiat.

[11] Courchesne E, Campbell K, Solso S (2011). Brain growth across the life span in autism: age-specific changes in anatomical pathology. Brain Res.

[12] Deoni SC, Zinkstok JR, Daly E, Ecker C, Consortium MA, Williams SC (2015). White-matter relaxation time and myelin water fraction differences in young adults with autism. Psychol Med.

[13] Phan BN, Bohlen JF, Davis BA, Ye Z, Chen HY, Mayfield B (2020). A myelin-related transcriptomic profile is shared by Pitt-Hopkins syndrome models and human autism spectrum disorder. Nat Neurosci.

[14] Gozzi M, Nielson DM, Lenroot RK, Ostuni JL, Luckenbaugh DA, Thurm AE (2012). A magnetization transfer imaging study of corpus callosum myelination in young children with autism. Biol Psychiat.

[15] Khanbabaei M, Hughes E, Ellegood J, Qiu LR, Yip R, Dobry J (2019). Precocious myelination in a mouse model of autism. Transl Psychiatry.

[16] Pacey LK, Xuan IC, Guan S, Sussman D, Henkelman RM, Chen Y (2013). Delayed myelination in a mouse model of fragile X syndrome. Hum Mol Genet.

[17] Leblond CS, Nava C, Polge A, Gauthier J, Huguet G, Lumbroso S (2014). Meta-analysis of SHANK Mutations in Autism Spectrum Disorders: a gradient of severity in cognitive impairments. PLoS Genet.

[18] Costales JL, Kolevzon A (2015). Phelan-McDermid Syndrome and SHANK3: implications for Treatment. Neurotherapeutics : the journal of the American Society for Experimental NeuroTherapeutics.

[19] Sarasua SM, Boccuto L, Sharp JL, Dwivedi A, Chen CF, Rollins JD (2014). Clinical and genomic evaluation of 201 patients with Phelan-McDermid syndrome. Hum Genet.

[20] Bonaglia MC, Giorda R, Borgatti R, Felisari G, Gagliardi C, Selicorni A (2001). Disruption of the ProSAP2 gene in a t(12;22)(q24.1;q13.3) is associated with the 22q13.3 deletion syndrome. Am J Hum Genet.

[21] Betancur C, Buxbaum JD (2013). SHANK3 haploinsufficiency: a "common" but underdiagnosed highly penetrant monogenic cause of autism spectrum disorders. Mol Autism.

[22] Boeckers TM, Kreutz MR, Winter C, Zuschratter W, Smalla KH, Sanmarti-Vila L (1999). Proline-rich synapse-associated protein-1/cortactin binding protein 1 (ProSAP1/CortBP1) is a PDZ-domain protein highly enriched in the postsynaptic density. J Neurosci.

[23] Sheng M, Kim E (2000). The Shank family of scaffold proteins. J Cell Sci.

[24] Boeckers TM, Bockmann J, Kreutz MR, Gundelfinger ED (2002). ProSAP/Shank proteins—a family of higher order organizing molecules of the postsynaptic density with an emerging role in human neurological disease. J Neurochem.

[25] Sarowar T, Grabrucker AM (2016). Actin-dependent alterations of dendritic spine morphology in Shankopathies. Neural Plast.

[26] Boeckers TM (2006). The postsynaptic density. Cell Tissue Res.

[27] Grabrucker AM, Schmeisser MJ, Schoen M, Boeckers TM (2011). Postsynaptic ProSAP/Shank scaffolds in the cross-hair of synaptopathies. Trends Cell Biol.

[28] Grabrucker S, Proepper C, Mangus K, Eckert M, Chhabra R, Schmeisser MJ (2014). The PSD protein ProSAP2/Shank3 displays synapto-nuclear shuttling which is deregulated in a schizophrenia-associated mutation. Exp Neurol.

[29] Schoen M, Asoglu H, Bauer HF, Muller HP, Abaei A, Sauer AK (2019). Shank3 transgenic and prenatal zinc-deficient autism mouse models show convergent and individual alterations of brain structures in MRI. Front Neural Circuits.

[30] Jesse S, Muller HP, Schoen M, Asoglu H, Bockmann J, Huppertz HJ (2019). Severe white matter damage in SHANK3 deficiency: a human and translational study. Ann Clin Transl Neurol.

[31] Bassell J, Srivastava S, Prohl AK, Scherrer B, Kapur K, Filip-Dhima R (2020). Diffusion tensor imaging abnormalities in the uncinate fasciculus and inferior longitudinal fasciculus in Phelan-McDermid Syndrome. Pediatr Neurol.

[32] Schmeisser MJ, Ey E, Wegener S, Bockmann J, Stempel AV, Kuebler A (2012). Autistic-like behaviours and hyperactivity in mice lacking ProSAP1/Shank2. Nature.

[33] Lancaster MA, Knoblich JA (2014). Generation of cerebral organoids from human pluripotent stem cells. Nat Protoc.

[34] Allen Institute for Brain Science. Allen Brain Atlas API. 2007

[35] Franklin KBJ, Paxinos G (2007). The mouse brain in stereotaxic coordinates.

[36] Lutz AK, Pfaender S, Incearap B, Ioannidis V, Ottonelli I, Fohr KJ (2020). Autism-associated SHANK3 mutations impair maturation of neuromuscular junctions and striated muscles. Sci Transl Med..

[37] Giandomenico SL, Mierau SB, Gibbons GM, Wenger LMD, Masullo L, Sit T (2019). Cerebral organoids at the air-liquid interface generate diverse nerve tracts with functional output. Nat Neurosci.

[38] Medina-Rodriguez EM, Arenzana FJ, Bribian A, de Castro F (2013). Protocol to isolate a large amount of functional oligodendrocyte precursor cells from the cerebral cortex of adult mice and humans. PLoS ONE.

[39] Damstra HGJ, Mohar B, Eddison M, Akhmanov A, Kapitein LC, Tillberg PW. Visualizing cellular and tissue ultrastructure using Ten-fold Robust Expansion Microscopy (TREx). bioRxiv. bioRxiv2021. 10.7554/eLife.73775 PMC8887890 35179128

[40] van Tilborg E, van Kammen CM, de Theije CGM, van Meer MPA, Dijkhuizen RM, Nijboer CH (2017). A quantitative method for microstructural analysis of myelinated axons in the injured rodent brain. Sci Rep.

[41] Arancibia-Carcamo IL, Ford MC, Cossell L, Ishida K, Tohyama K, Attwell D (2017). Node of Ranvier length as a potential regulator of myelinated axon conduction speed. Elife.

[42] Schneider S, Gruart A, Grade S, Zhang Y, Kroger S, Kirchhoff F (2016). Decrease in newly generated oligodendrocytes leads to motor dysfunctions and changed myelin structures that can be rescued by transplanted cells. Glia.

[43] Orefice LL, Mosko JR, Morency DT, Wells MF, Tasnim A, Mozeika SM (2019). Targeting Peripheral Somatosensory Neurons to Improve Tactile-Related Phenotypes in ASD Models. Cell.

[44] Raasakka A, Kursula P. How Does Protein Zero Assemble Compact Myelin? Cells. 2020;9(8). doi: 10.3390/cells9081832. 10.3390/cells9081832 PMC7465998 32759708

[45] Galvez-Contreras AY, Zarate-Lopez D, Torres-Chavez AL, Gonzalez-Perez O (2020). Role of oligodendrocytes and myelin in the pathophysiology of autism spectrum disorder. Brain Sci.

[46] Courchesne E, Pierce K (2005). Brain overgrowth in autism during a critical time in development: implications for frontal pyramidal neuron and interneuron development and connectivity. Int J Dev Neurosci.

[47] Noriuchi M, Kikuchi Y, Yoshiura T, Kira R, Shigeto H, Hara T (2010). Altered white matter fractional anisotropy and social impairment in children with autism spectrum disorder. Brain Res.

[48] Deoni SC, Dean DC, O'Muircheartaigh J, Dirks H, Jerskey BA (2012). Investigating white matter development in infancy and early childhood using myelin water faction and relaxation time mapping. Neuroimage.

[49] Wolff JJ, Gerig G, Lewis JD, Soda T, Styner MA, Vachet C (2015). Altered corpus callosum morphology associated with autism over the first 2 years of life. Brain.

[50] Zhou Y, Sharma J, Ke Q, Landman R, Yuan J, Chen H (2019). Atypical behaviour and connectivity in SHANK3-mutant macaques. Nature.

[51] Golden CEM, Wang VX, Harony-Nicolas H, Hof PR, Buxbaum JD (2021). Reduced brain volume and white matter alterations in Shank3-deficient rats. Autism Res.

[52] Reichova A, Bacova Z, Bukatova S, Kokavcova M, Meliskova V, Frimmel K (2020). Abnormal neuronal morphology and altered synaptic proteins are restored by oxytocin in autism-related SHANK3 deficient model. Mol Cell Endocrinol.

[53] Huang G, Chen S, Chen X, Zheng J, Xu Z, Doostparast Torshizi A (2019). Uncovering the Functional Link Between SHANK3 Deletions and Deficiency in Neurodevelopment Using iPSC-Derived Human Neurons. Front Neuroanat.

[54] Liu C, Li D, Yang H, Li H, Xu Q, Zhou B (2021). Altered striatum centered brain structures in SHANK3 deficient Chinese children with genotype and phenotype profiling. Prog Neurobiol.

[55] Jin C, Kang H, Ryu JR, Kim S, Zhang Y, Lee Y (2018). Integrative brain transcriptome analysis reveals region-specific and broad molecular changes in Shank3-overexpressing mice. Front Mol Neurosci.

[56] Lee Y, Kang H, Lee B, Zhang Y, Kim Y, Kim S (2017). Integrative analysis of brain region-specific Shank3 interactomes for understanding the heterogeneity of neuronal pathophysiology related to SHANK3 mutations. Front Mol Neurosci.

[57] Lee Y, Kim SG, Lee B, Zhang Y, Kim Y, Kim S (2017). Striatal Transcriptome and Interactome Analysis of Shank3-overexpressing Mice Reveals the Connectivity between Shank3 and mTORC1 Signaling. Front Mol Neurosci.

[58] Quadrato G, Nguyen T, Macosko EZ, Sherwood JL, Min Yang S, Berger DR (2017). Cell diversity and network dynamics in photosensitive human brain organoids. Nature.

[59] Skoff RP (1990). Gliogenesis in rat optic nerve: astrocytes are generated in a single wave before oligodendrocytes. Dev Biol.

[60] Barres BA, Raff MC (1999). Axonal control of oligodendrocyte development. J Cell Biol.

[61] Soorya L, Kolevzon A, Zweifach J, Lim T, Dobry Y, Schwartz L (2013). Prospective investigation of autism and genotype-phenotype correlations in 22q13 deletion syndrome and SHANK3 deficiency. Molecular autism.

[62] Bonaglia MC, Giorda R, Beri S, De Agostini C, Novara F, Fichera M (2011). Molecular mechanisms generating and stabilizing terminal 22q13 deletions in 44 subjects with Phelan/McDermid syndrome. PLoS Genet.

[63] Philippe A, Craus Y, Rio M, Bahi-Buisson N, Boddaert N, Malan V (2015). Case report: an unexpected link between partial deletion of the SHANK3 gene and Heller's dementia infantilis, a rare subtype of autism spectrum disorder. BMC Psychiatry.

[64] Figura MG, Coppola A, Bottitta M, Calabrese G, Grillo L, Luciano D (2014). Seizures and EEG pattern in the 22q13.3 deletion syndrome: clinical report of six Italian cases. Seizure..

[65] Graciarena M, Seiffe A, Nait-Oumesmar B, Depino AM (2018). Hypomyelination and oligodendroglial alterations in a mouse model of autism spectrum disorder. Front Cell Neurosci.

[66] Ju J, Yang X, Jiang J, Wang D, Zhang Y, Zhao X (2021). Structural and lipidomic alterations of striatal myelin in 16p112 deletion mouse model of autism spectrum disorder. Front Cell Neurosci.

[67] Aggarwal S, Snaidero N, Pahler G, Frey S, Sanchez P, Zweckstetter M (2013). Myelin membrane assembly is driven by a phase transition of myelin basic proteins into a cohesive protein meshwork. PLoS Biol.

[68] Phelan MC (2008). Deletion 22q13.3 syndrome. Orphanet J Rare Dis.

[69] Phelan K, McDermid HE (2012). The 22q13.3 Deletion Syndrome (Phelan-McDermid Syndrome). Mol Syndromol..

[70] Reierson G, Bernstein J, Froehlich-Santino W, Urban A, Purmann C, Berquist S (2017). Characterizing regression in Phelan McDermid Syndrome (22q13 deletion syndrome). J Psychiatr Res.

[71] Kohlenberg TM, Trelles MP, McLarney B, Betancur C, Thurm A, Kolevzon A (2020). Psychiatric illness and regression in individuals with Phelan-McDermid syndrome. J Neurodev Disord.

[72] Cullen CL, Pepper RE, Clutterbuck MT, Pitman KA, Oorschot V, Auderset L (2021). Periaxonal and nodal plasticities modulate action potential conduction in the adult mouse brain. Cell Rep.

[73] Kidd GJ, Ohno N, Trapp BD (2013). Biology of Schwann cells. Handb Clin Neurol.

[74] Simons M, Trotter J (2007). Wrapping it up: the cell biology of myelination. Curr Opin Neurobiol.

[75] Siems SB, Jahn O, Eichel MA, Kannaiyan N, Wu LMN, Sherman DL (2020). Proteome profile of peripheral myelin in healthy mice and in a neuropathy model. Elife.

[76] Readhead C, Popko B, Takahashi N, Shine HD, Saavedra RA, Sidman RL (1987). Expression of a myelin basic protein gene in transgenic shiverer mice: correction of the dysmyelinating phenotype. Cell.

[77] Smith-Slatas C, Barbarese E (2000). Myelin basic protein gene dosage effects in the PNS. Mol Cell Neurosci.

[78] Martini R, Mohajeri MH, Kasper S, Giese KP, Schachner M (1995). Mice doubly deficient in the genes for P0 and myelin basic protein show that both proteins contribute to the formation of the major dense line in peripheral nerve myelin. J Neurosci.

[79] Lemke G, Axel R (1985). Isolation and sequence of a cDNA encoding the major structural protein of peripheral myelin. Cell.

[80] Arancibia-Carcamo IL, Attwell D (2014). The node of Ranvier in CNS pathology. Acta Neuropathol.

