# Supplementary material for: SHANK3 deficiency leads to myelin defects in the central and peripheral nervous system
Source: Cell Mol Life Sci. 2022 Jun 20;79(7):371. doi: 10.1007/s00018-022-04400-4 (PMC9209365; doi:10.1007/s00018-022-04400-4)

Supplementary Fig. 1

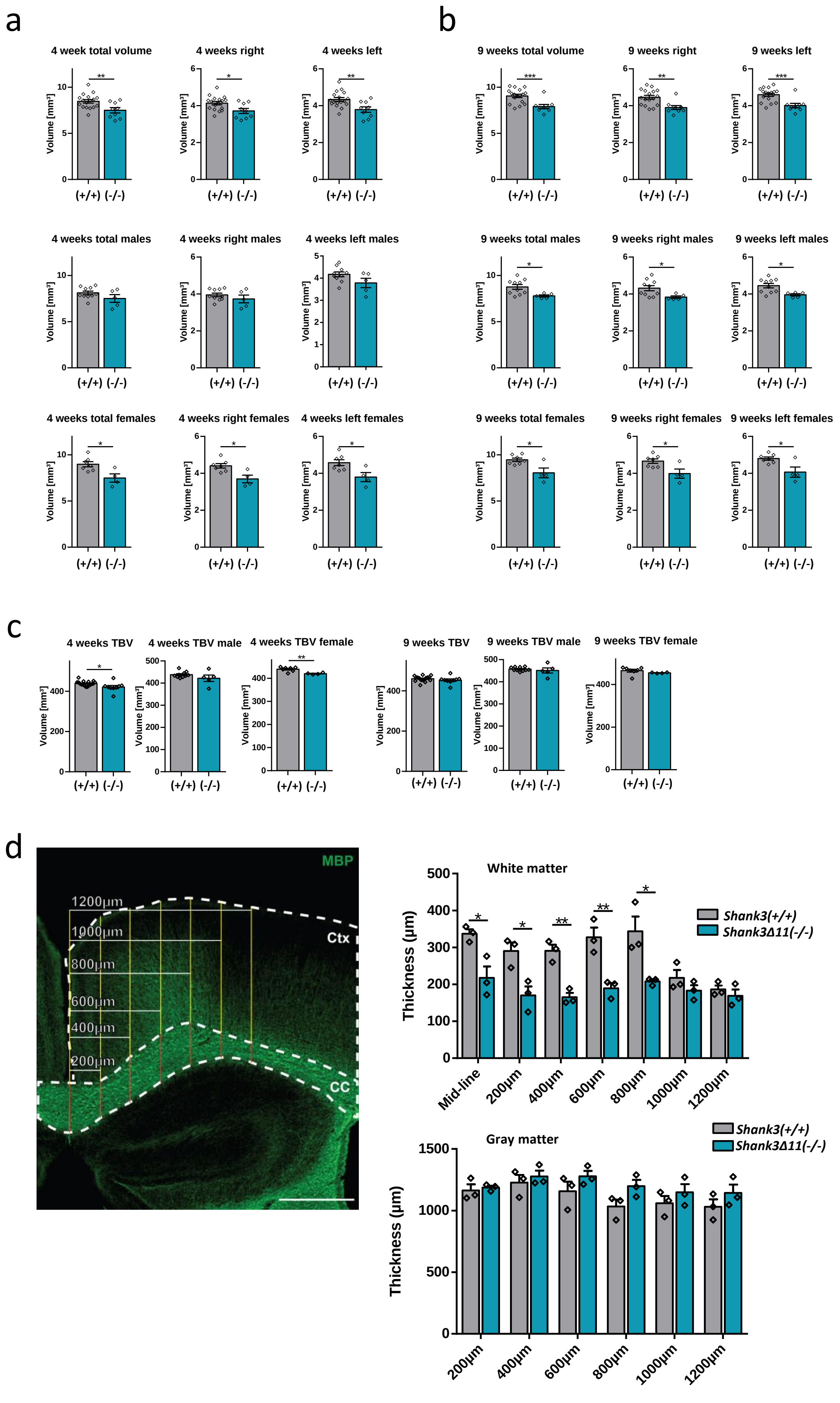

Supplementary Fig. 2

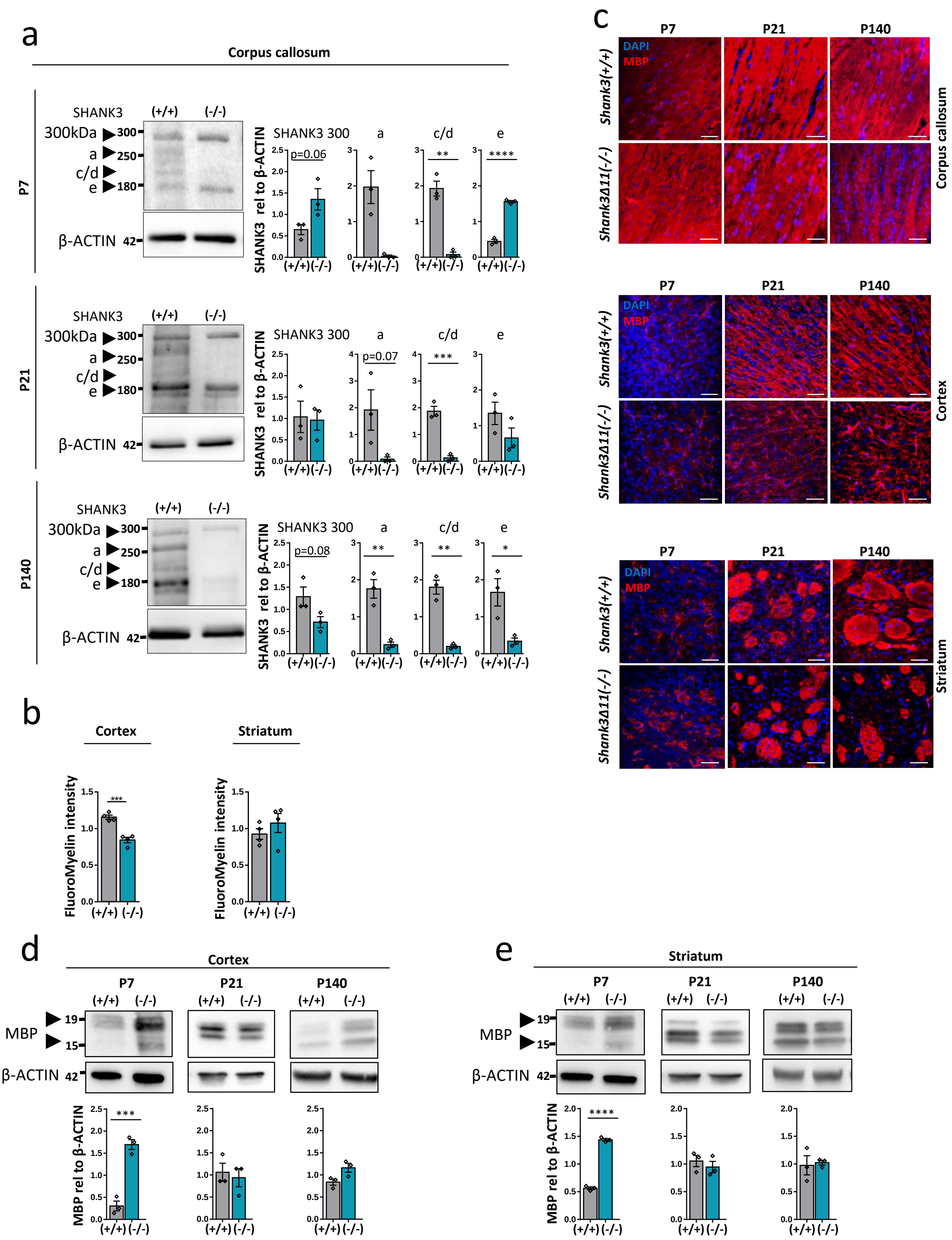

Supplementary Fig. 3

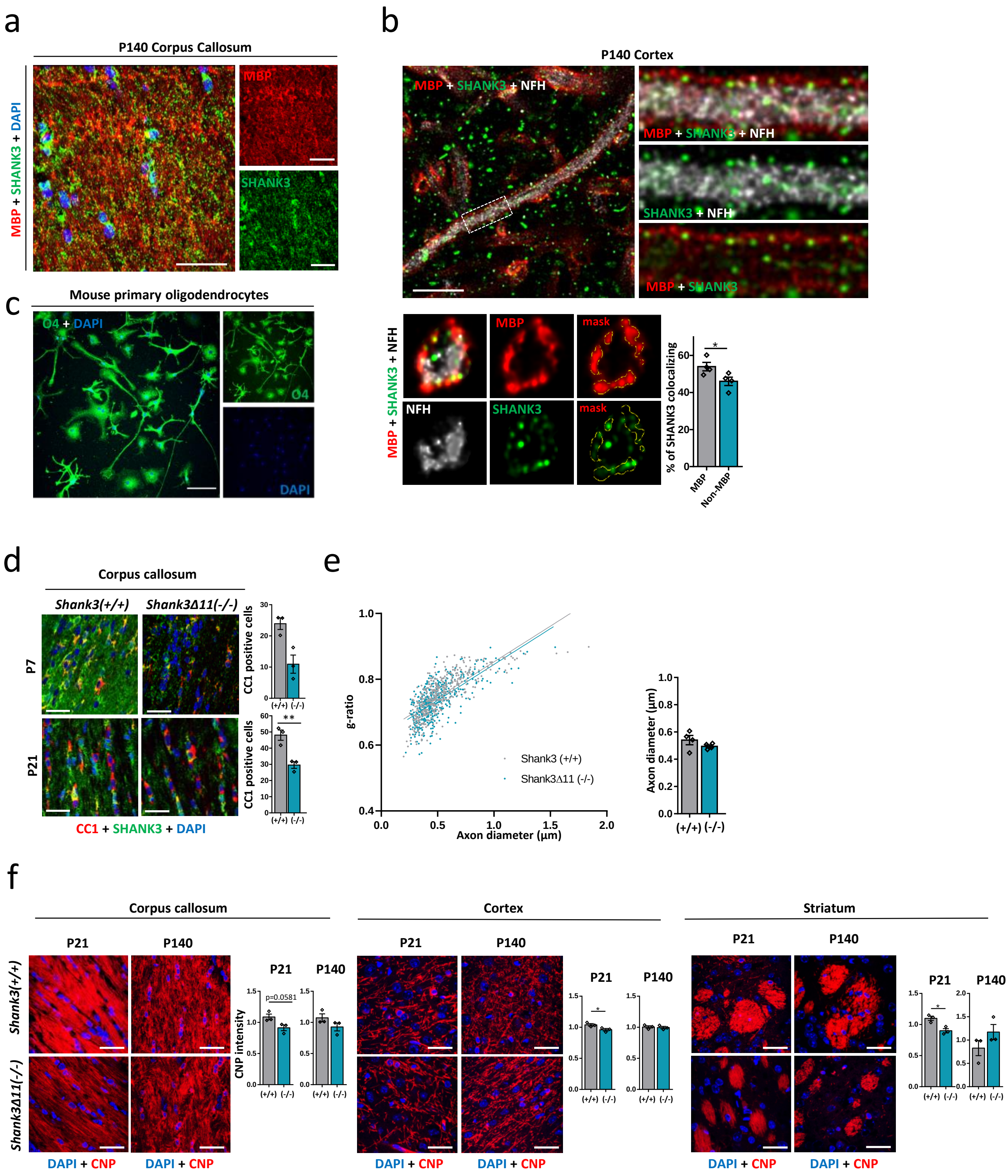

Supplementary Fig. 4

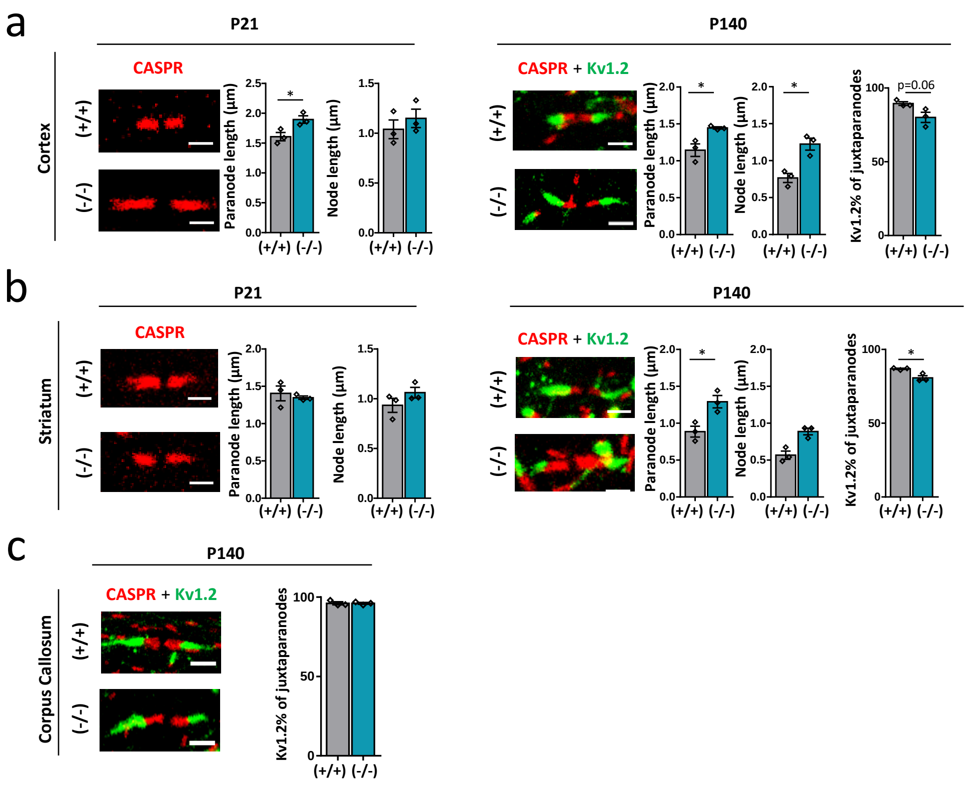

Supplementary Fig. 5

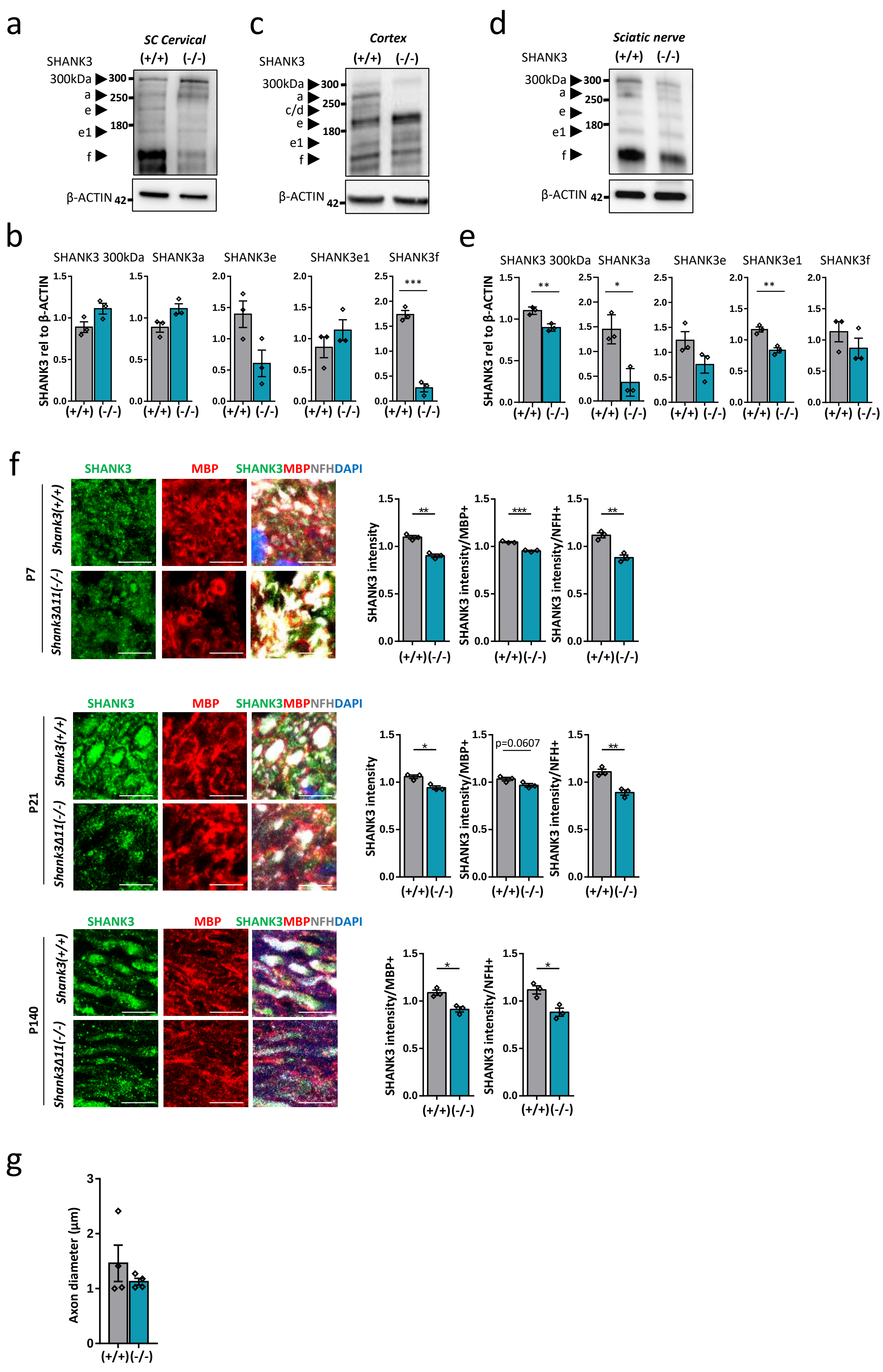

Supplementary Fig. 6

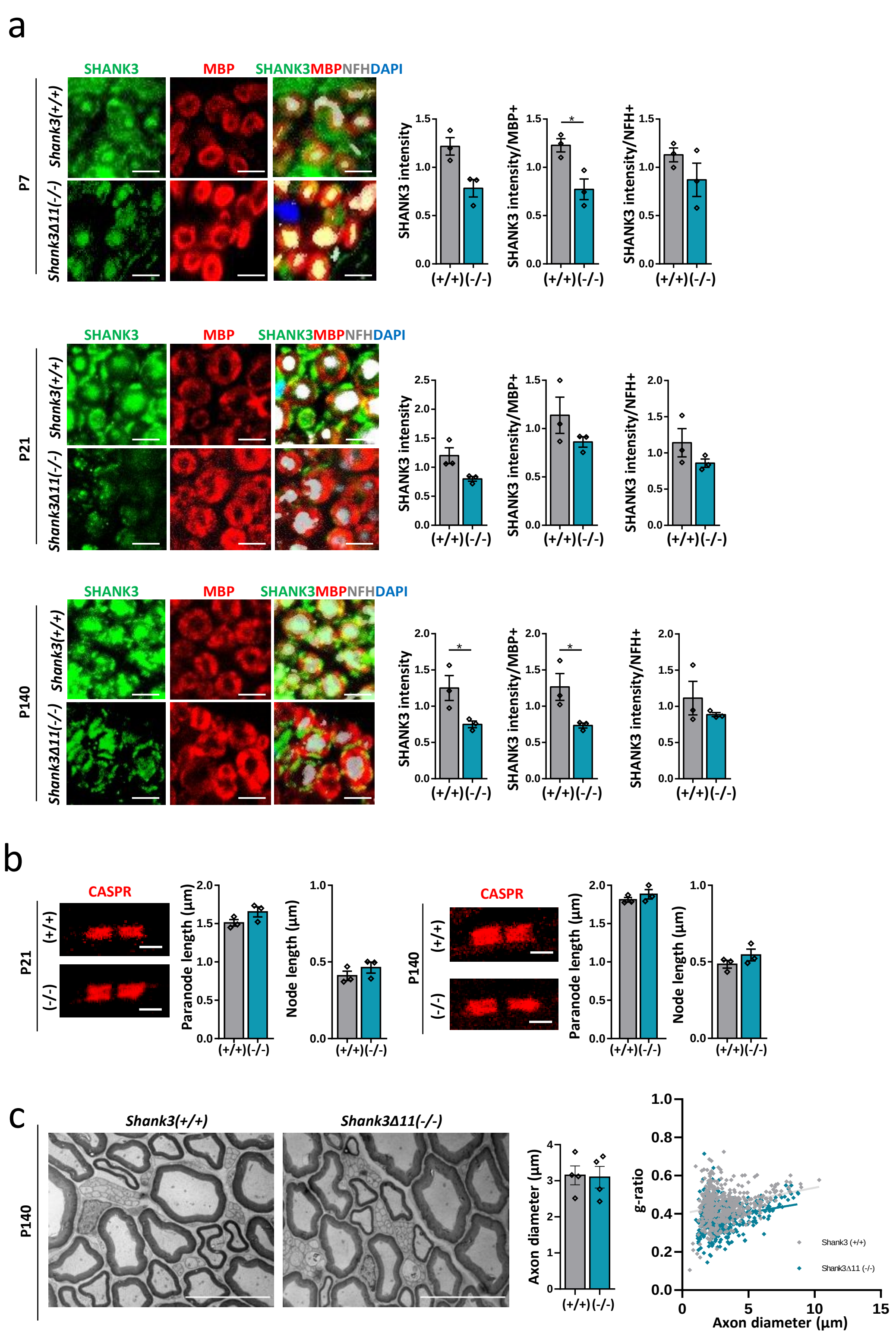

Supplement: Supplementary file 1 — Supplementary Figure 1. a MRI analysis of corpus callosum volume in 4-week-old Shank3(+/+) and Shank3Δ11(−/−) animals. Males: n=10 +/+, n=5 −/− animals. Females: n=7 +/+, n=4 −/− animals Mean±SEM. Student’s Unpaired t-test, total p=0.0075, right p=0.0153, left p=0.0048, total males p=0.1523, right males p=0.2914, left males p=0.0892, total females p=0.0151, right females p=0.0106, left females p=0.0212. b MRI analysis of corpus callosum volume in 9-week-old Shank3(+/+) and Shank3Δ11(−/−) animals. n=10 +/+, n=5 −/− animals. Mean±SEM. Student’s Unpaired t-test, total p=0.0009, right p=0.0020, left p=0.0006, total males p=0.0211, right males p=0.0333, left males p=0.0149, total females p=0.0132, right females p=0.0206, left females p=0.0119. c Total brain volume analysis in 4-week-old Shank3(+/+) and Shank3Δ11(−/−) animals. n=10 +/+, n=5 −/− animals. Mean±SEM. Student’s Unpaired t-test, total p=0.0156, males p=0.1843, females p=0.0046. Total brain volume analysis in 9-week-old Shank3(+/+) and Shank3Δ11(−/−) animals. n=10 +/+, n=5 −/− animals. Mean±SEM. Student’s Unpaired t-test, total p=0.2736, males p=0.5036, females p=0.2798. d MBP IHC and analysis of gray and white matter thickness of 140-day-old Shank3(+/+) and Shank3Δ11(−/−) animals. Scale bar 500μm. n=3 animals. Mean±SEM. Student’s Unpaired t-test. Corpus callosum: distance was measured from the mid-line up to 1200μm. Mid-line: p=0.0226, 200μm p=0.0222, 400μm p=0.0033, 600μm p=0.0098, 800μm p=0.0272, 1000μm p=0.2496. 1200μm p=0.4404. Cortex: 200μm p=0.6818, 400μm p=0.5539, 600μm p=0.2489, 800μm p=0.0953, 1000μm p=0.3735, 1200μm p=0.2858. Supplementary Figure 2. a Western Blot analysis of SHANK3 and β-ACTIN in corpus callosum of 7-, 21- and 140-day-old Shank3(+/+) and Shank3Δ11(−/−) animals. n=3 animals. Mean±SEM. Student’s Unpaired t-test. P7: 300kDa p=0.0628, c/d p=0.0011, e p<0.0001. Mann-Whitney test. P7: a p=0.1000 P21: 300kDa p=0.8707, a p=0.00734, c/d p=0.0009, e p=0.1764; P140: 300kDa p=0.0831, a p=0.0044, [file 18_2022_4400_MOESM1_ESM.pdf]
